# Supplementary material for: Interpretable spatially aware dimension reduction of spatial transcriptomics with STAMP
Source: Nat Methods. 2024 Oct 15;21(11):2072–83. doi: 10.1038/s41592-024-02463-8 (PMC11541207; doi:10.1038/s41592-024-02463-8)
Supplement: Supplementary file 1 — Supplementary Figs. 1–24, Supplementary Notes 1 and 2 and Supplementary Tables 1 and 2. [file 41592_2024_2463_MOESM1_ESM.pdf]

nature methods

Supplementary information https://doi.org/10.1038/s41592-024-02463-8

Interpretable spatially aware dimension reduction of spatial transcriptomics with STAMP

In the format provided by the authors and unedited

Contents

Supplementary Figures 2
Supplementary Note 1: Technical details 23
Supplementary Note 2: Simulation study 26
Supplementary Tables 30
Supplementary References 32

Supplementary Figures

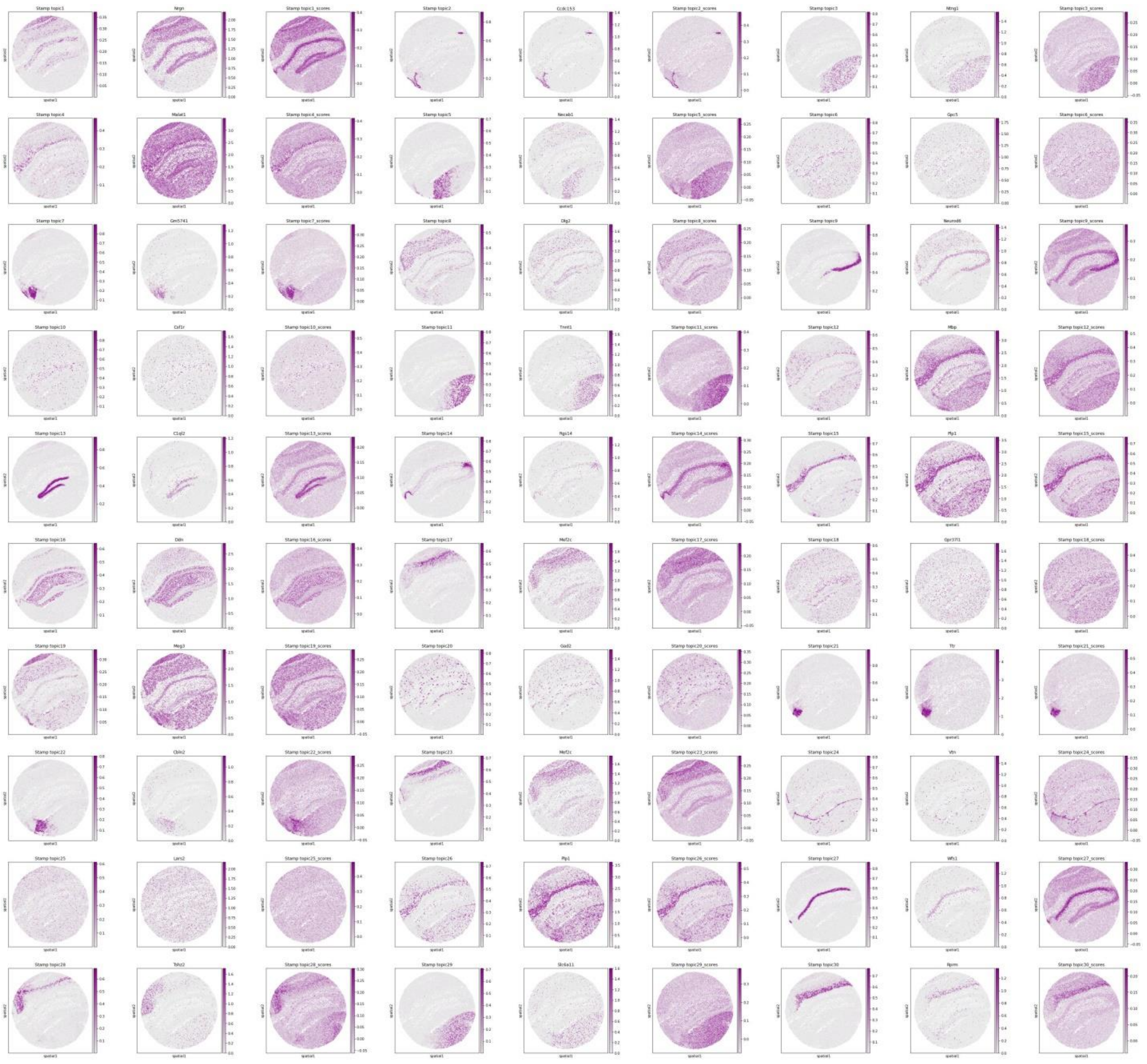

Figure S1: Topic proportion, normalized expression of the top gene and aggregated expression of the top 20 genes in the corresponding gene modules returned by STAMP for the mouse hippocampus Slide-seq V2 data. A similar pattern shared between the three plots means that the identified gene modules and the topic proportions are coherent.

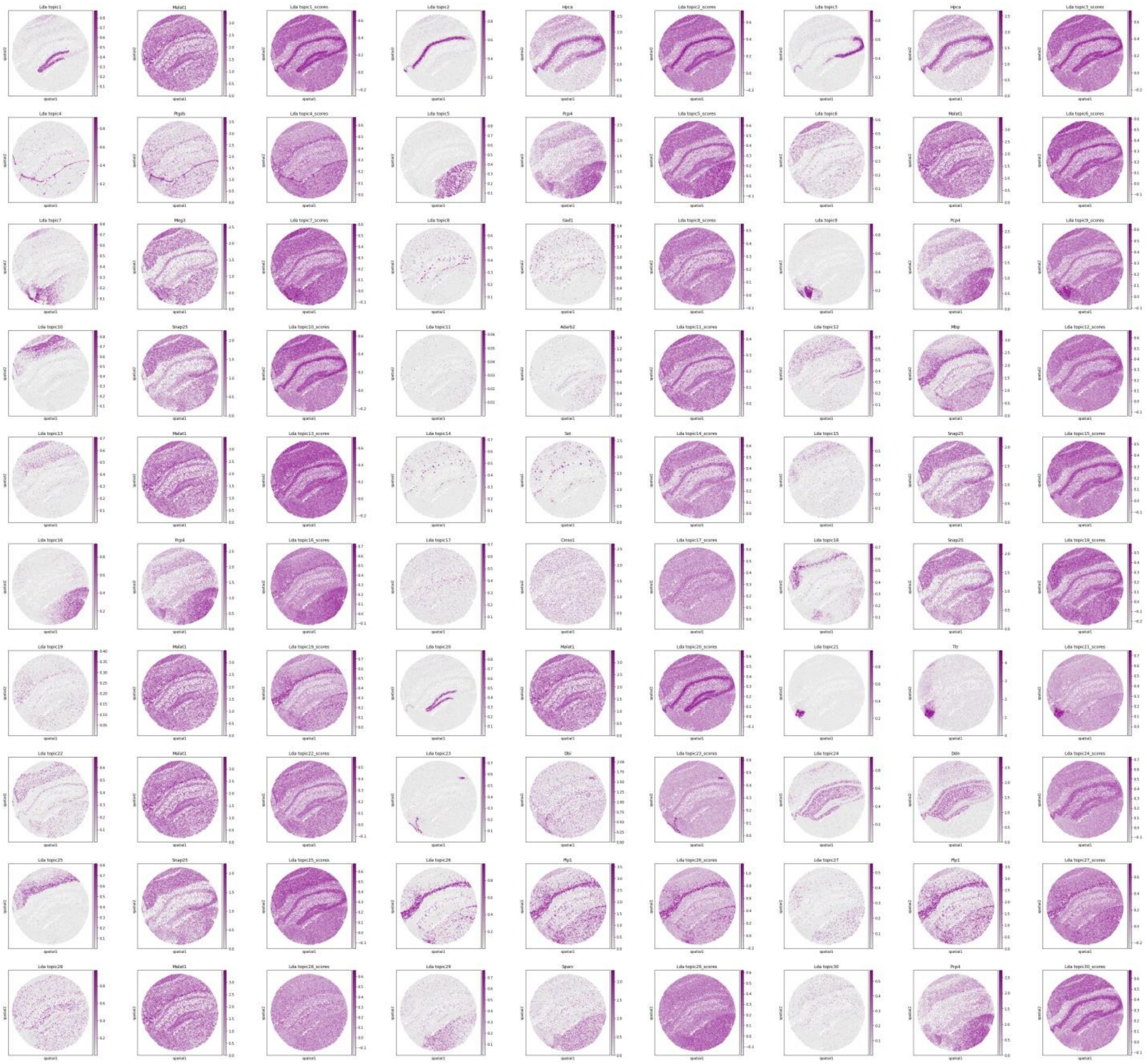

Figure S2: Topic proportion, normalized expression of the top gene and aggregated expression of the top 20 genes in the corresponding gene modules returned by LDA for the mouse hippocampus Slide-seq V2 data. A similar pattern shared between the three plots means that the identified gene modules and the topic proportions are coherent.

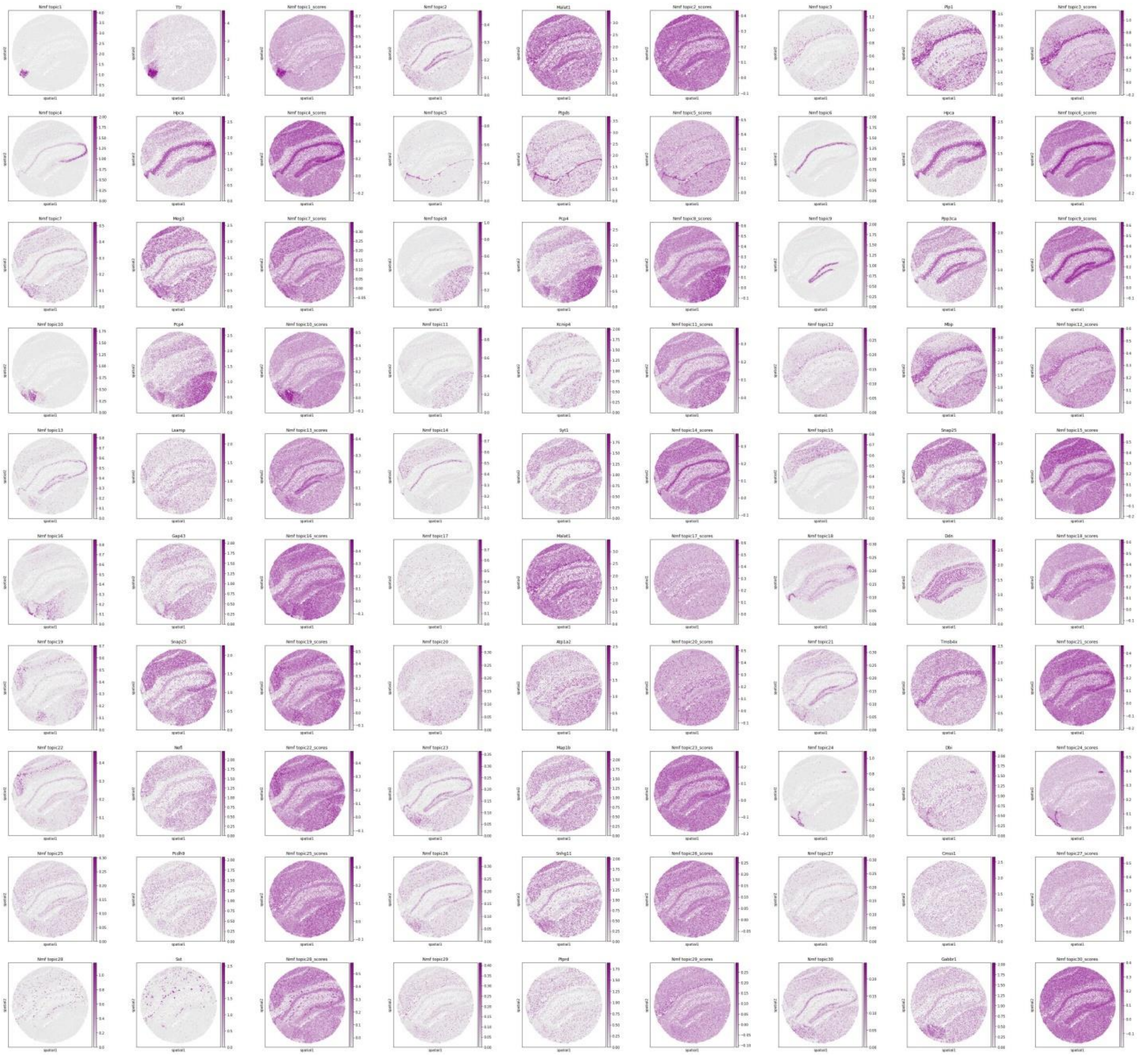

Figure S3: Topic proportion, normalized expression of the top gene and aggregated expression of the top 20 genes in the corresponding gene modules returned by NMF for the mouse hippocampus Slide-seq V2 data. A similar pattern shared between the three plots means that the identified gene modules and the topic proportions are coherent.

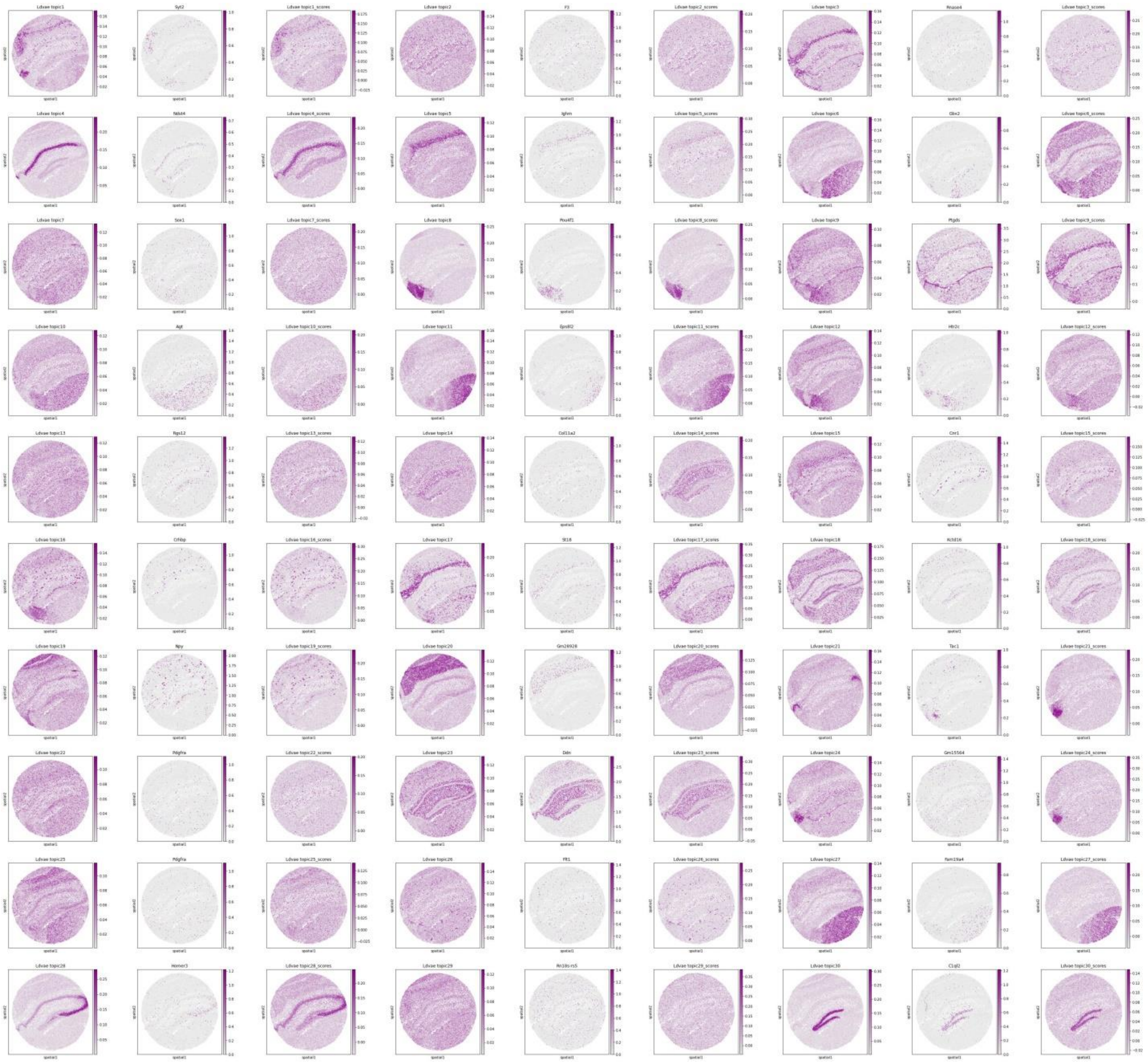

Figure S4: Topic proportion, normalized expression of the top gene and aggregated expression of the top 20 genes in the corresponding gene modules returned by LDVAE for the mouse hippocampus Slide-seq V2 data. A similar pattern shared between the three plots means that the identified gene modules and the topic proportions are coherent.

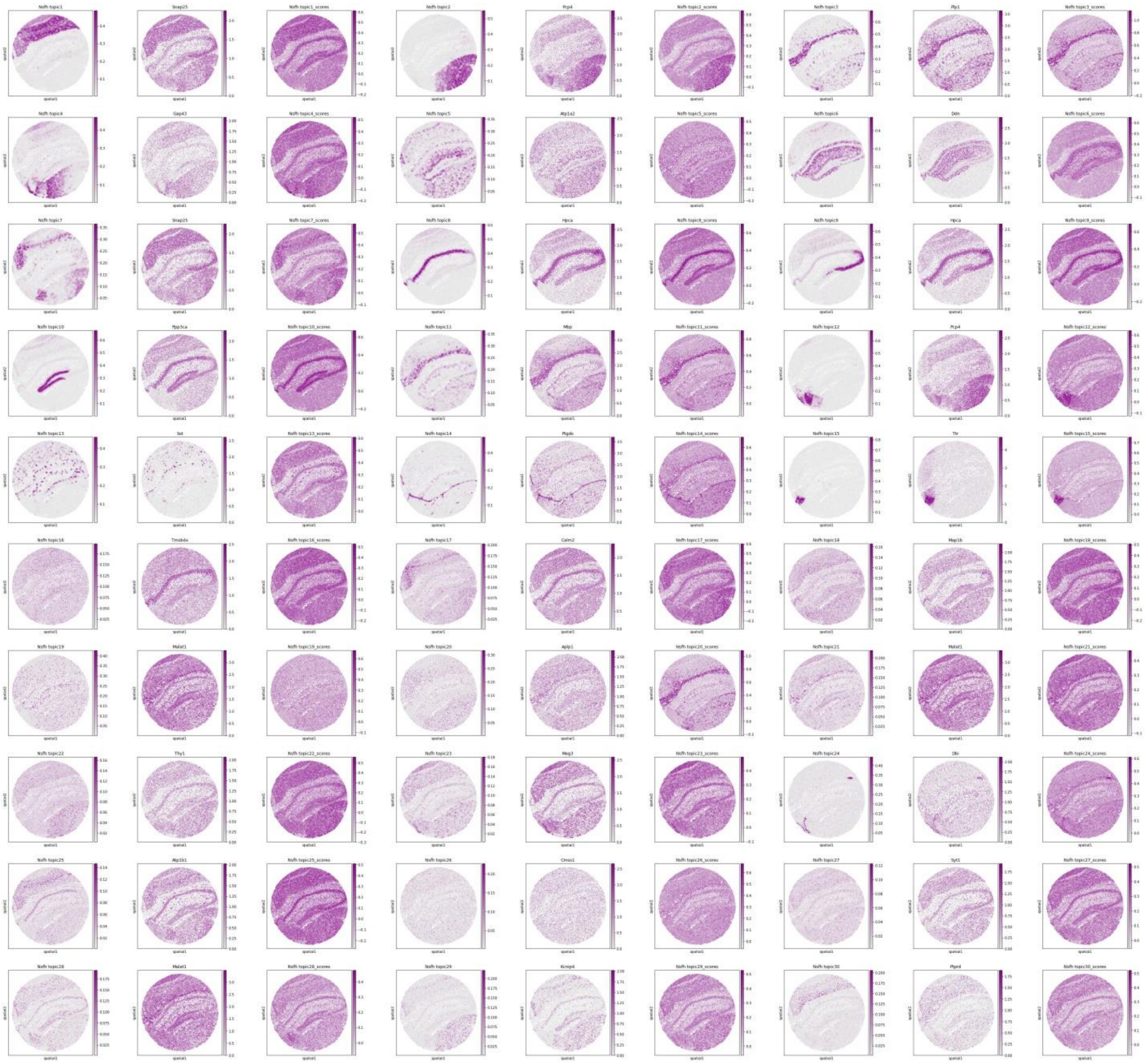

Figure S5: Topic proportion, normalized expression of the top gene and aggregated expression of the top 20 genes in the corresponding gene modules returned by NSFH for the mouse hippocampus Slide-seq V2 data. A similar pattern shared between the three plots means that the identified gene modules and the topic proportions are coherent.

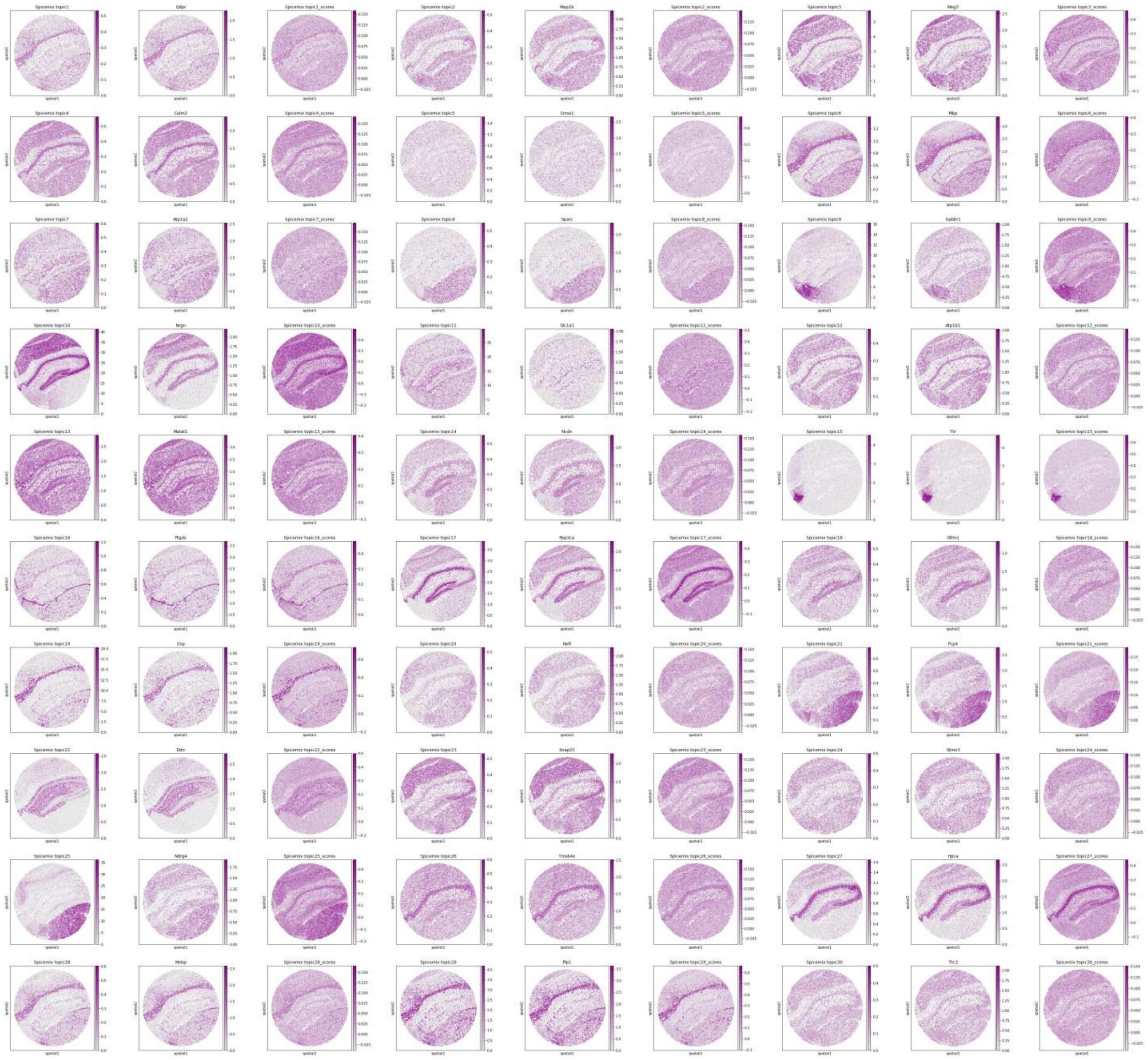

Figure S6: Topic proportion, normalized expression of the top gene and aggregated expression of the top 20 genes in the corresponding gene modules returned by SpiceMix for the mouse hippocampus Slide-seq V2 data. A similar pattern shared between the three plots means that the identified gene modules and the topic proportions are coherent.

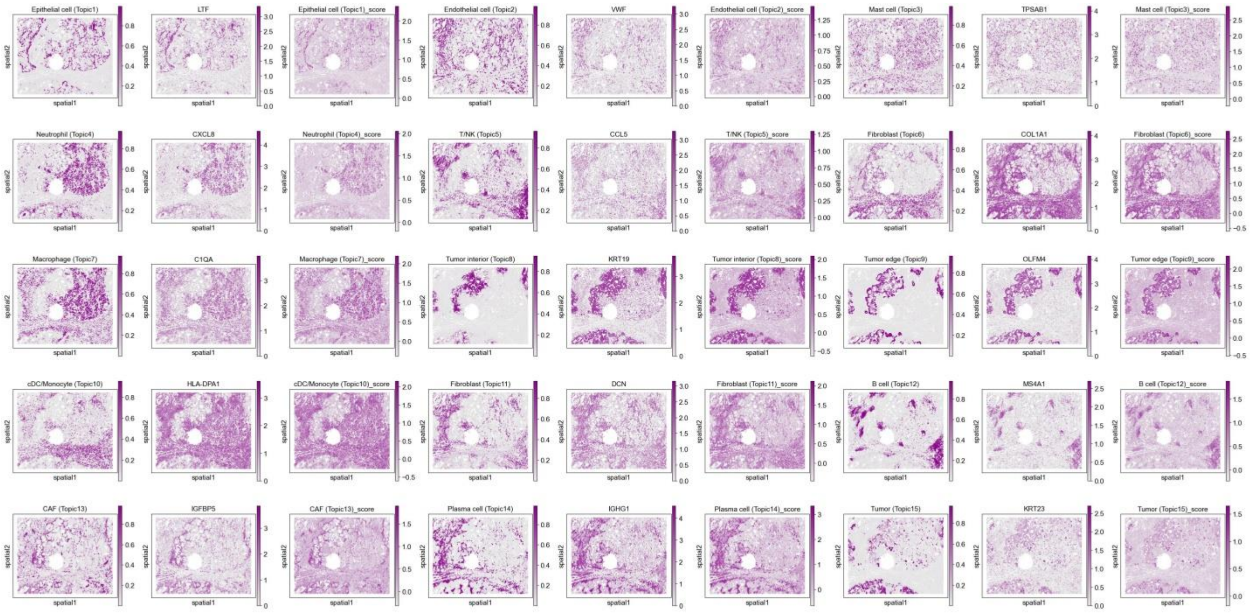

Figure S7: Topic proportion, normalized expression of the top gene and aggregated expression of the top 20 genes in the corresponding gene modules returned by STAMP for the Nanostring SMI NSCLC data. A similar pattern shared between the three plots means that the identified gene modules and the topic proportions are coherent.

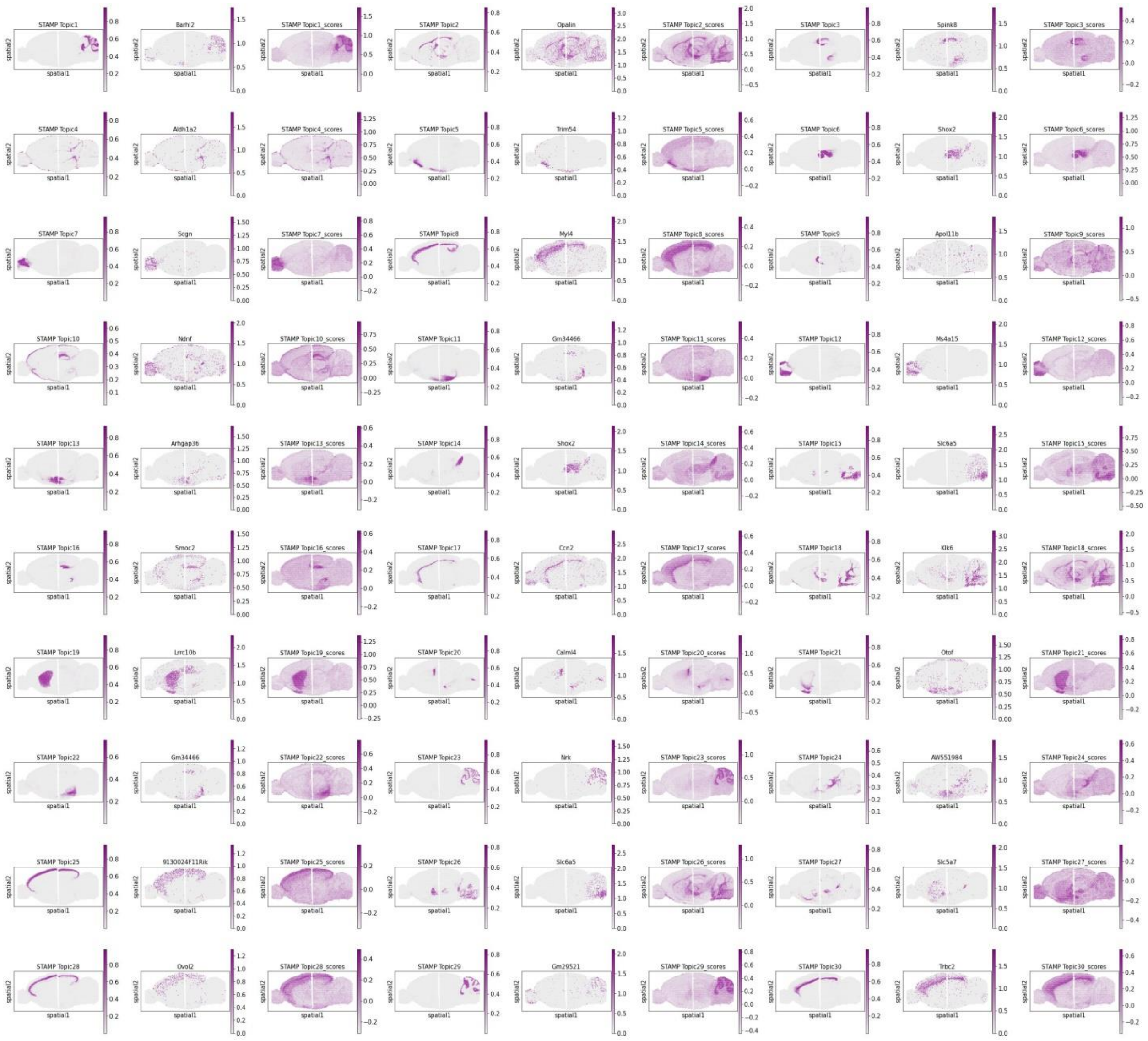

Figure S8: Topic proportion, normalized expression of the top gene and aggregated expression of the top 20 genes in the corresponding gene modules returned by STAMP for the 10x Genomics Visium mouse brain data. A similar pattern shared between the three plots means that the identified gene modules and the topic proportions are coherent.

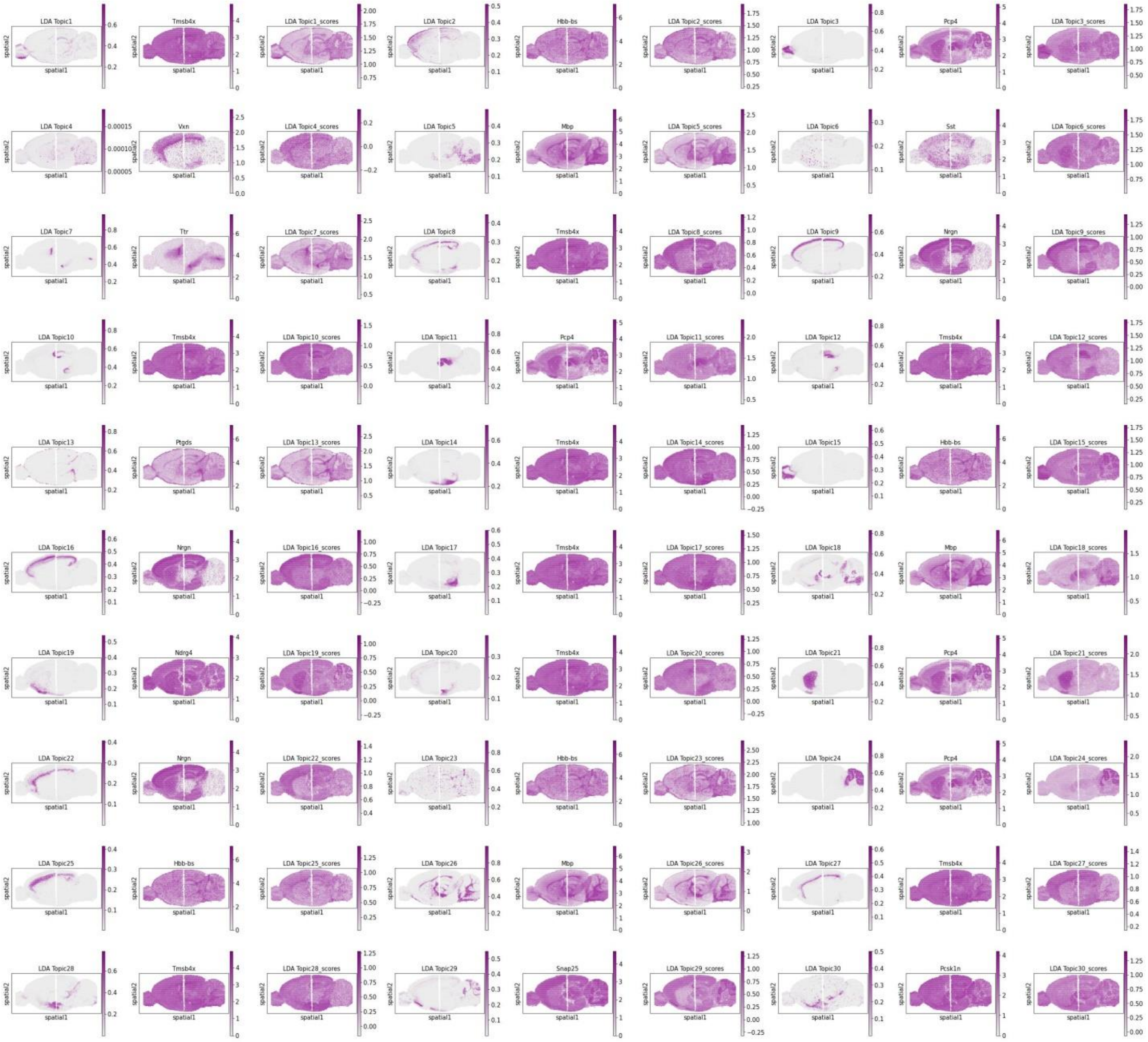

Figure S9: Topic proportion, normalized expression of the top gene and aggregated expression of the top 20 genes in the corresponding gene modules returned by LDA for the 10x Genomics Visium mouse brain data. A similar pattern shared between the three plots means that the identified gene modules and the topic proportions are coherent.

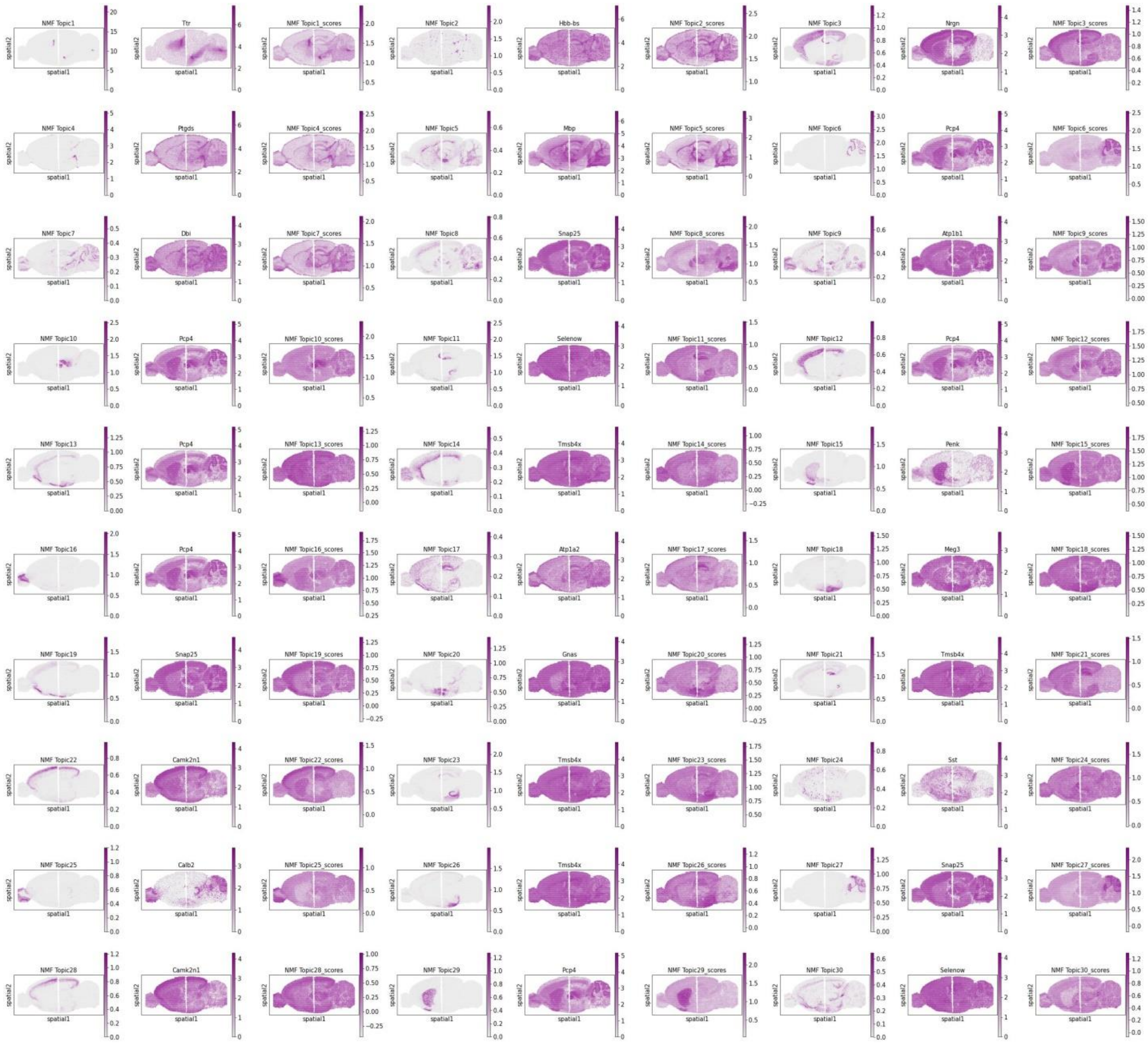

Figure S10: Topic proportion, normalized expression of the top gene and aggregated expression of the top 20 genes in the corresponding gene modules returned by NMF for the 10x Genomics Visium mouse brain data. A similar pattern shared between the three plots means that the identified gene modules and the topic proportions are coherent.

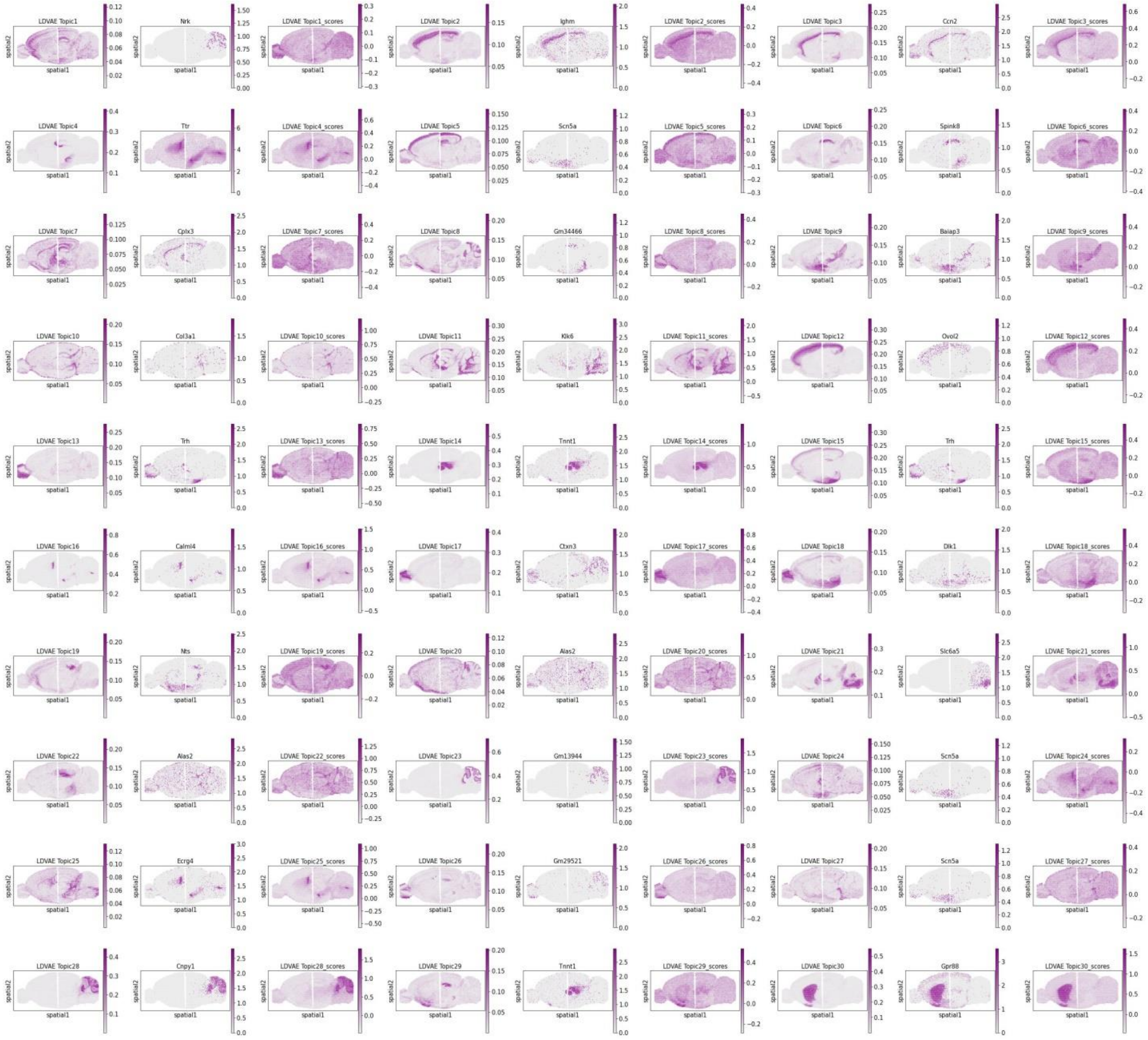

Figure S11: Topic proportion, normalized expression of the top gene and aggregated expression of the top 20 genes in the corresponding gene modules returned by LDVAE for the 10x Genomics Visium mouse brain data. A similar pattern shared between the three plots means that the identified gene modules and the topic proportions are coherent.

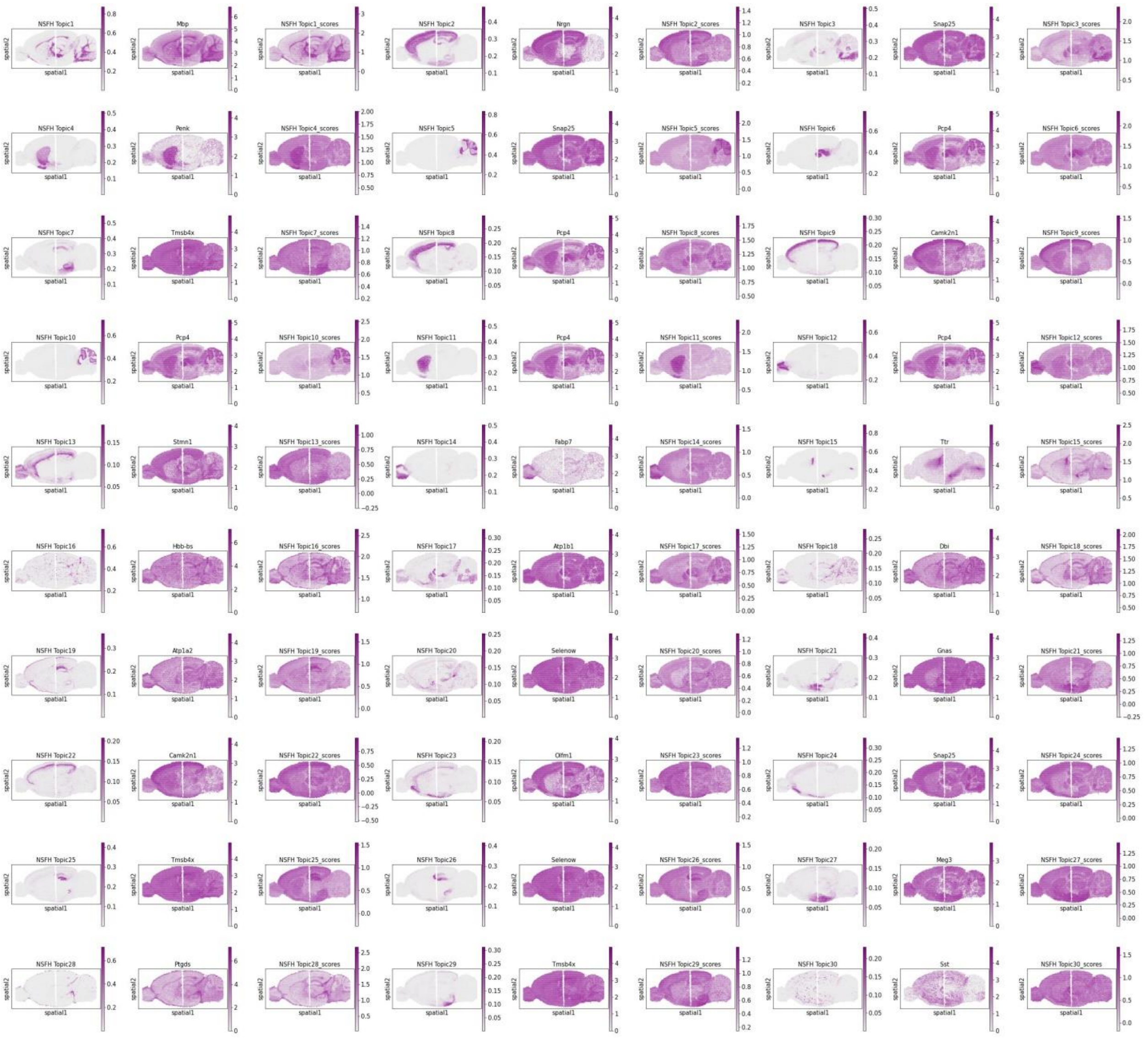

Figure S12: Topic proportion, normalized expression of the top gene and aggregated expression of the top 20 genes in the corresponding gene modules returned by NSFH for the 10x Genomics Visium mouse brain data. A similar pattern shared between the three plots means that the identified gene modules and the topic proportions are coherent.

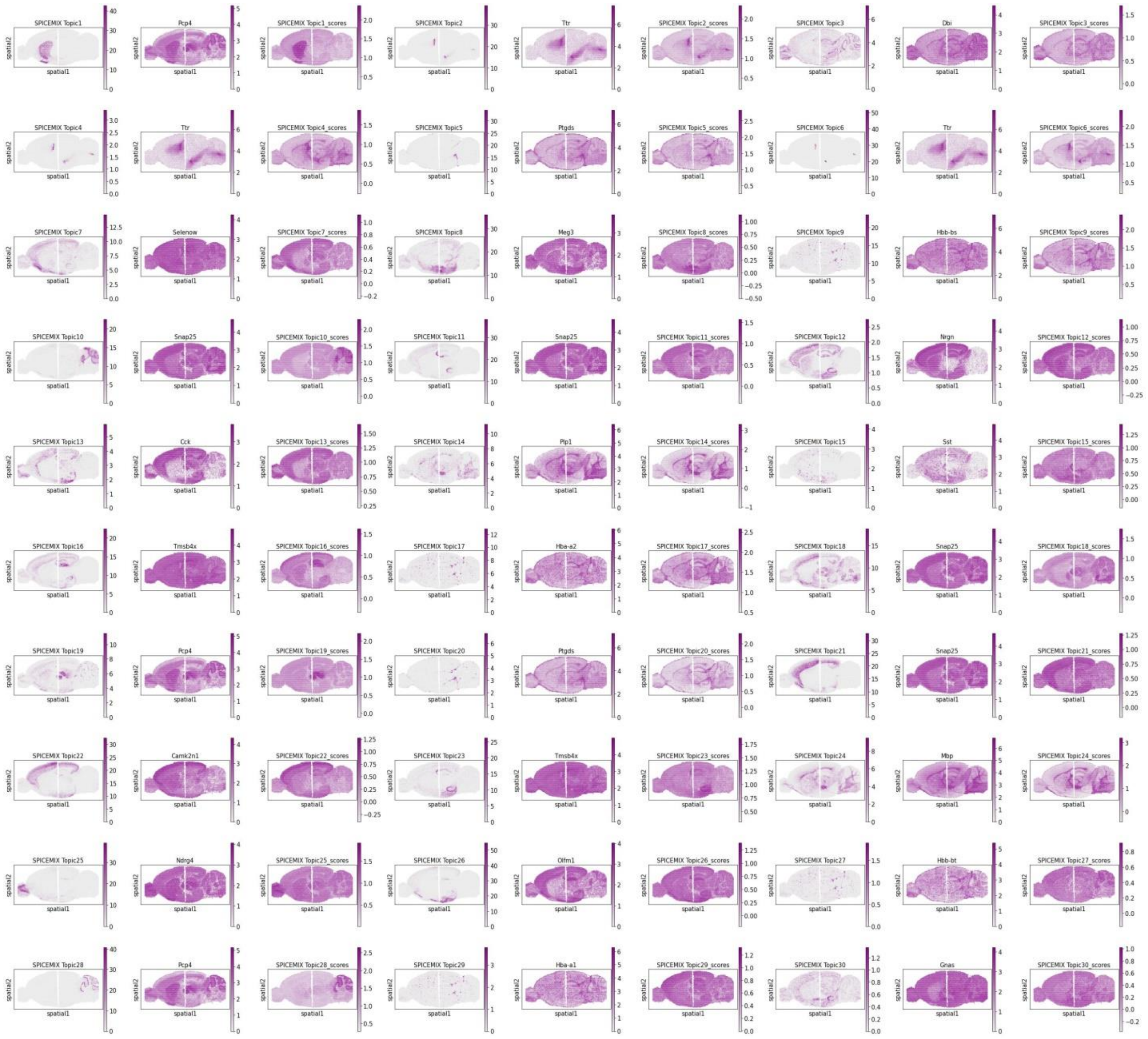

Figure S13: Topic proportion, normalized expression of the top gene and aggregated expression of the top 20 genes in the corresponding gene modules returned by SpiceMix for the 10x Genomics Visium mouse brain data. A similar pattern shared between the three plots means that the identified gene modules and the topic proportions are coherent.

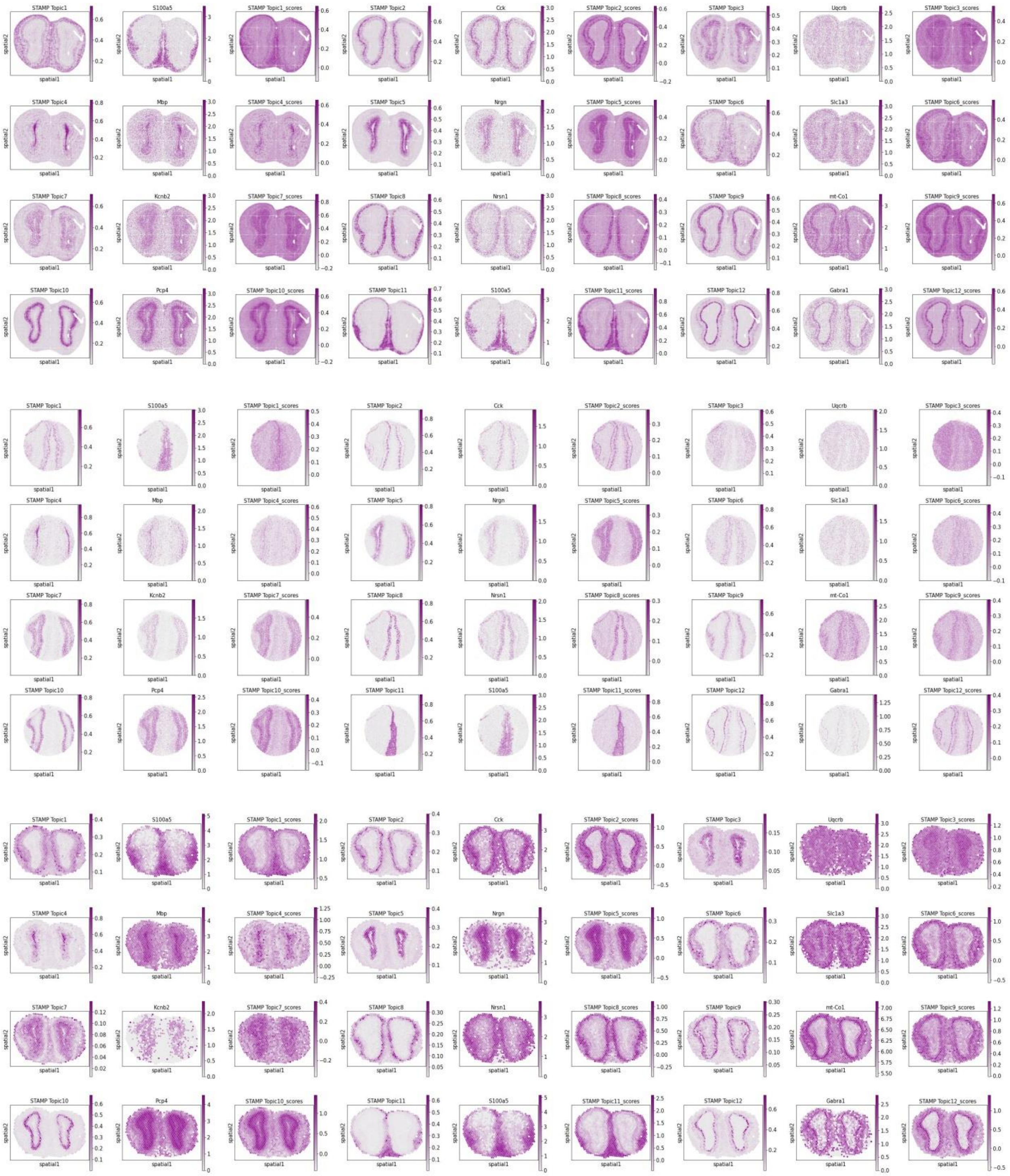

Figure S14: Topic proportion, normalized expression of the top gene and aggregated expression of the top 20 genes in the corresponding gene modules returned by STAMP for the Stereo-seq, Slide-seq, and Visium mouse olfactory bulb data. A similar pattern shared between the three plots means that the identified gene modules and the topic proportions are coherent.

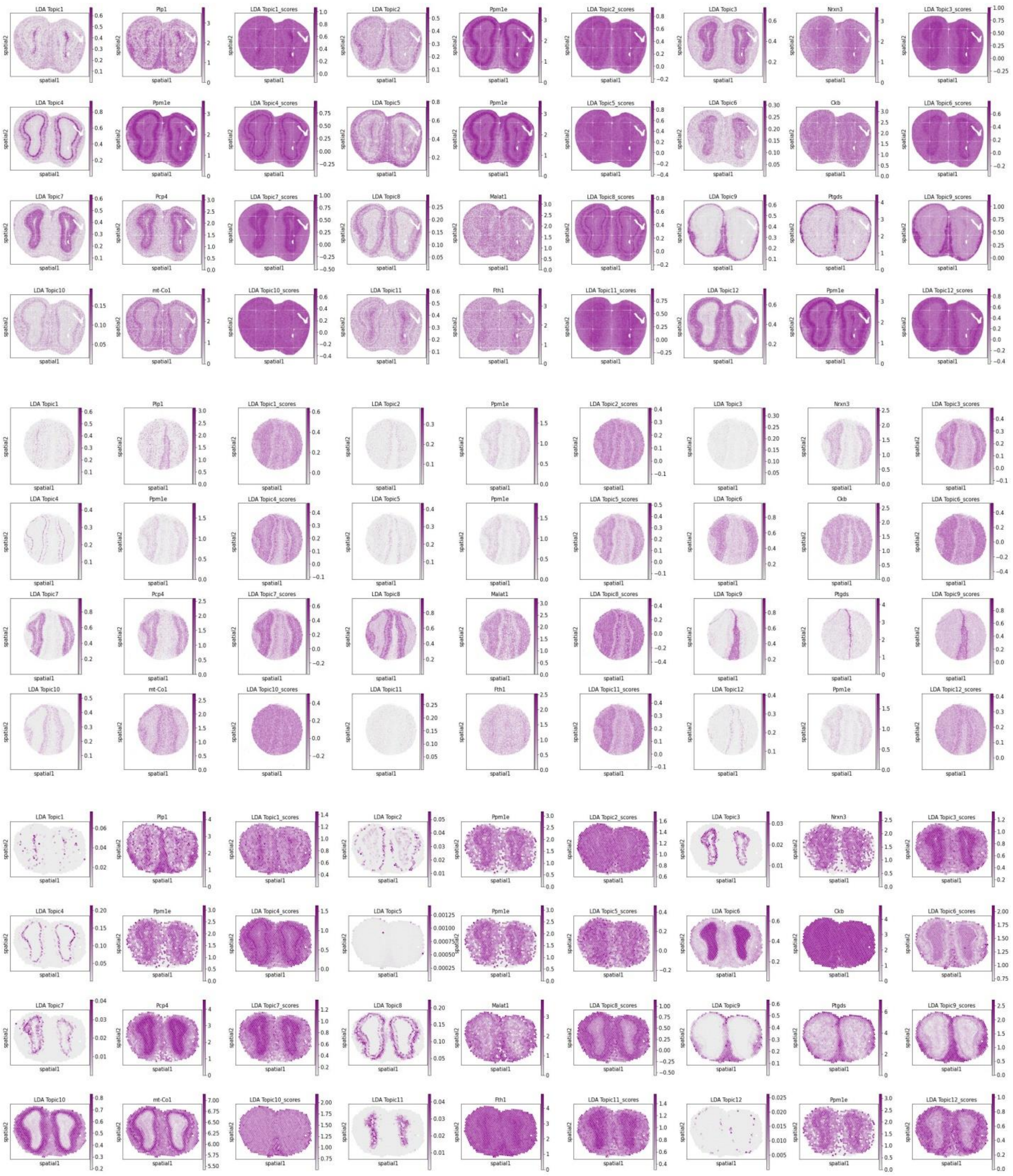

Figure S15: Topic proportion, normalized expression of the top gene and aggregated expression of the top 20 genes in the corresponding gene modules returned by LDA for the Stereo-seq, Slide-seq, and Visium mouse olfactory bulb data. A similar pattern shared between the three plots means that the identified gene modules and the topic proportions are coherent.

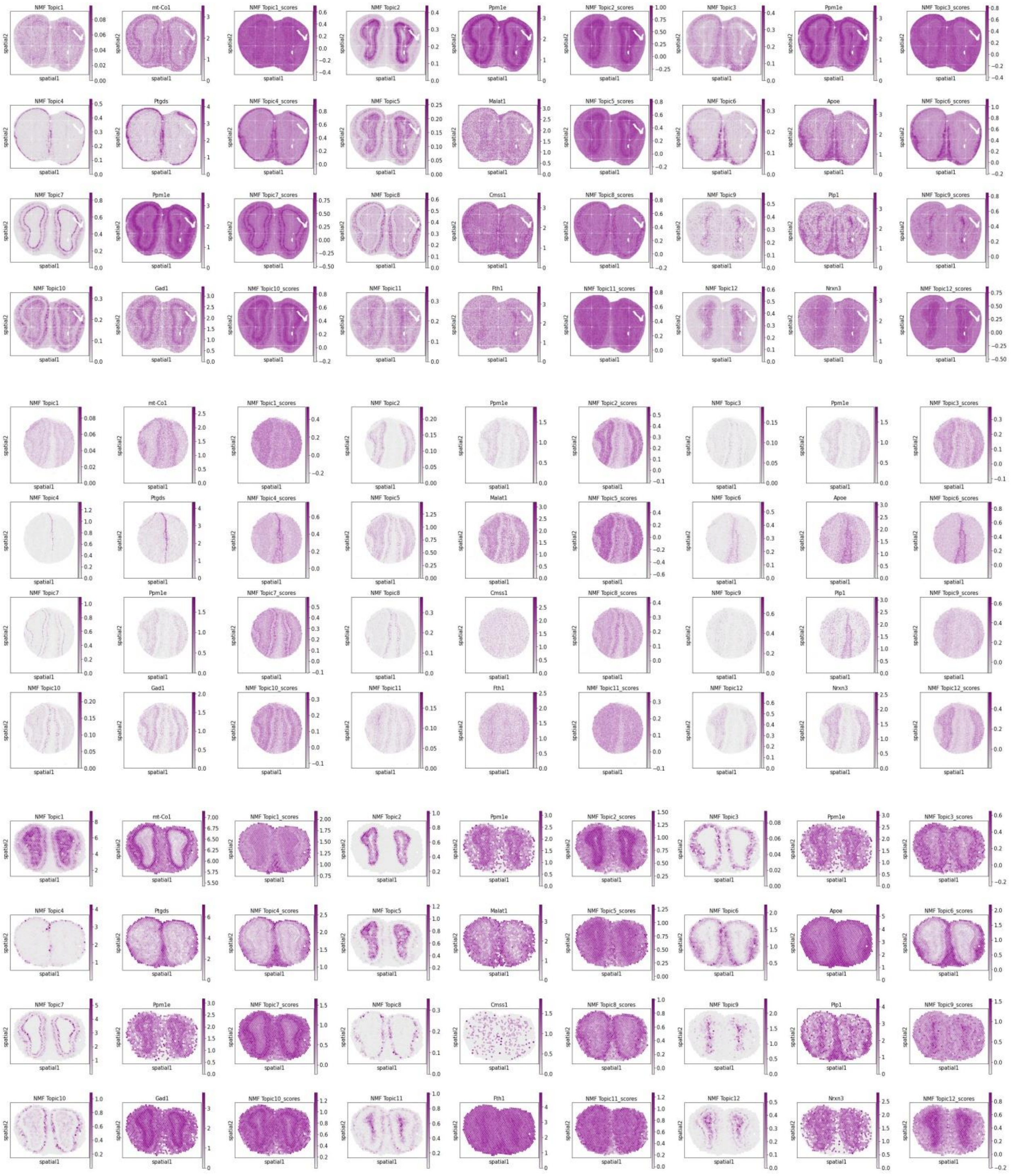

Figure S16: Topic proportion, normalized expression of the top gene and aggregated expression of the top 20 genes in the corresponding gene modules returned by NMF for the Stereo-seq, Slide-seq, and Visium mouse olfactory bulb data. A similar pattern shared between the three plots means that the identified gene modules and the topic proportions are coherent.

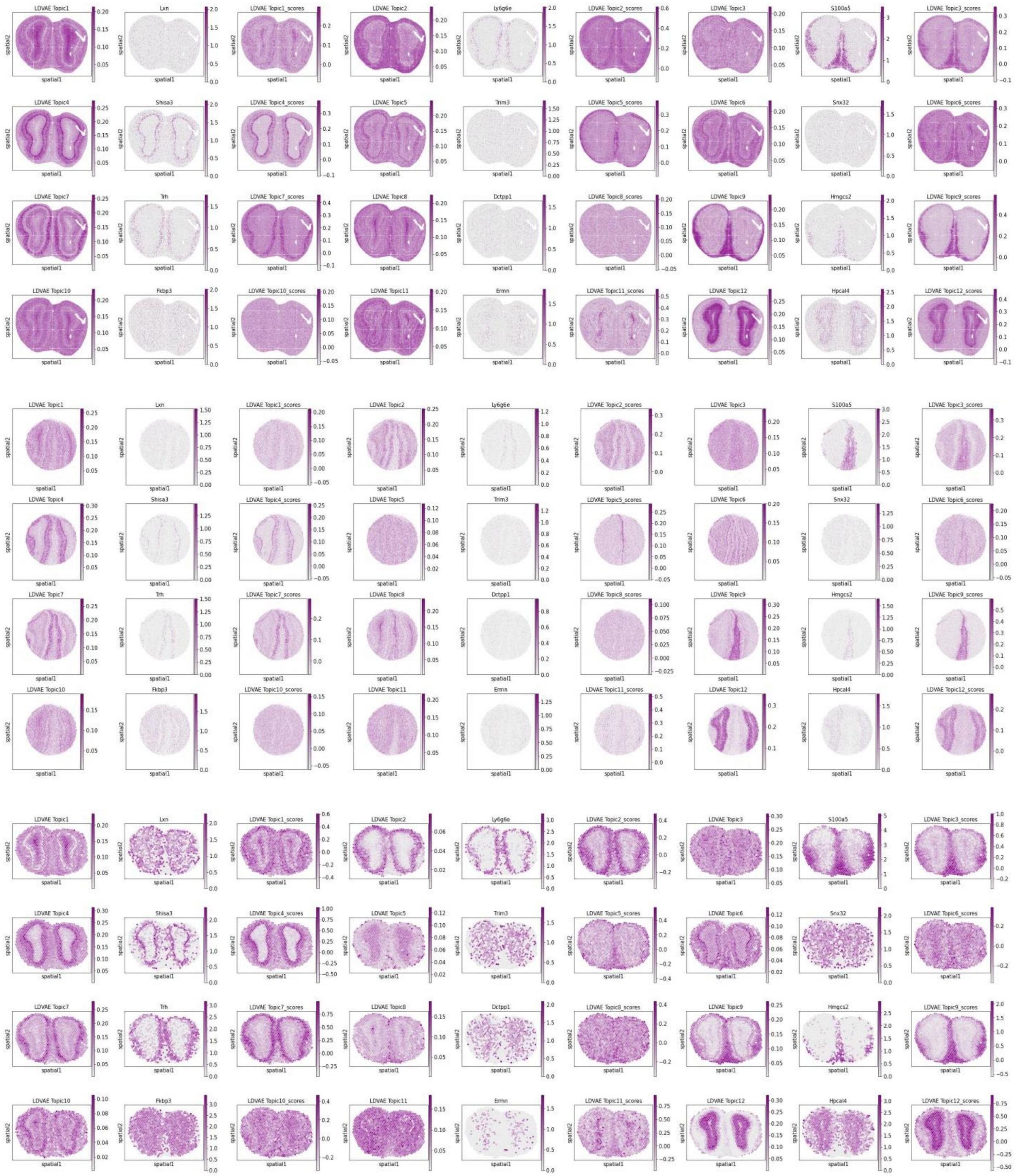

Figure S17: Topic proportion, normalized expression of the top gene and aggregated expression of the top 20 genes in the corresponding gene modules returned by LDVAE for the Stereo-seq, Slide-seq, and Visium mouse olfactory bulb data. A similar pattern shared between the three plots means that the identified gene modules and the topic proportions are coherent.

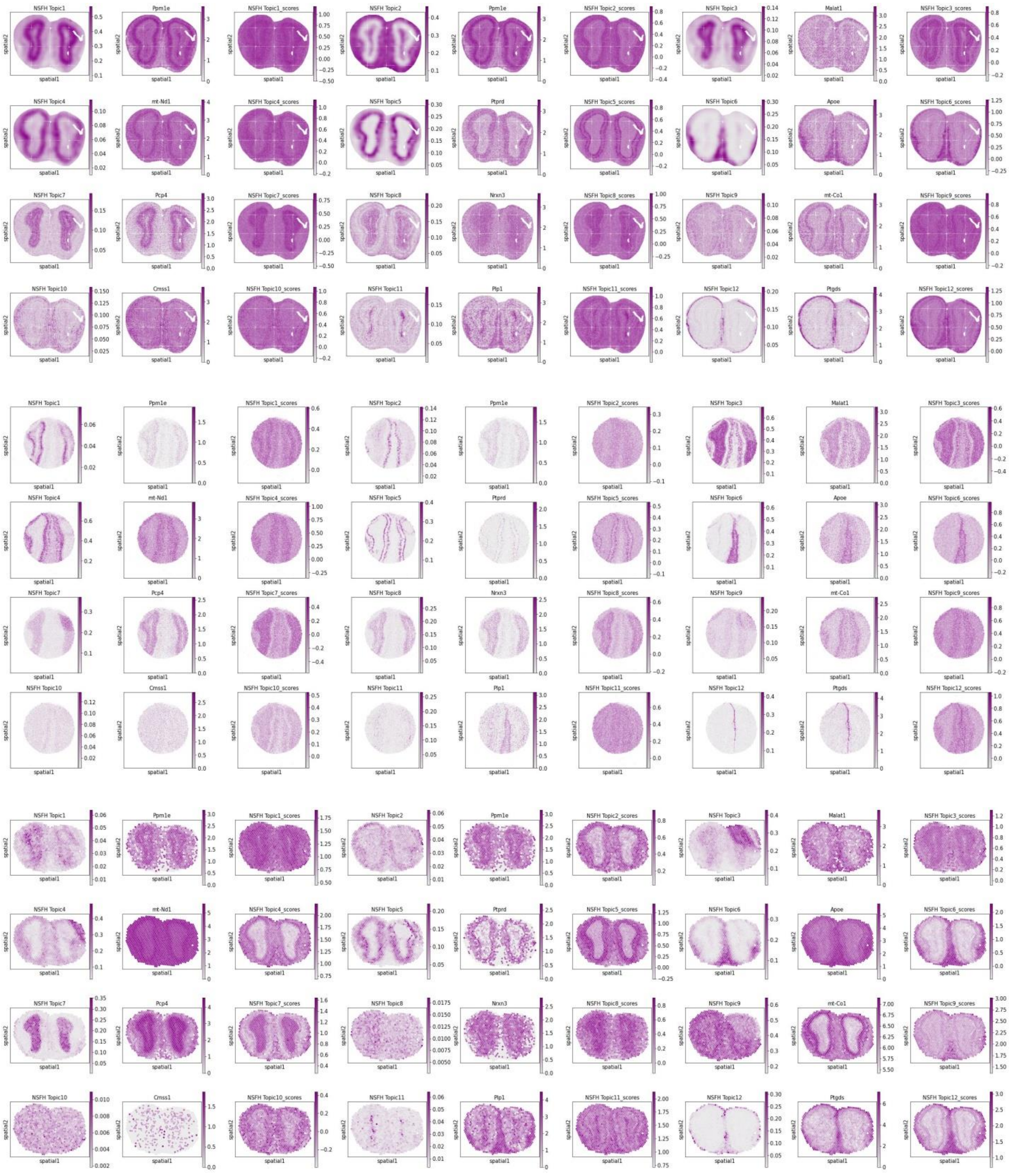

Figure S18: Topic proportion, normalized expression of the top gene and aggregated expression of the top 20 genes in the corresponding gene modules returned by NSFH for the Stereo-seq, Slide-seq, and Visium mouse olfactory bulb data. A similar pattern shared between the three plots means that the identified gene modules and the topic proportions are coherent.

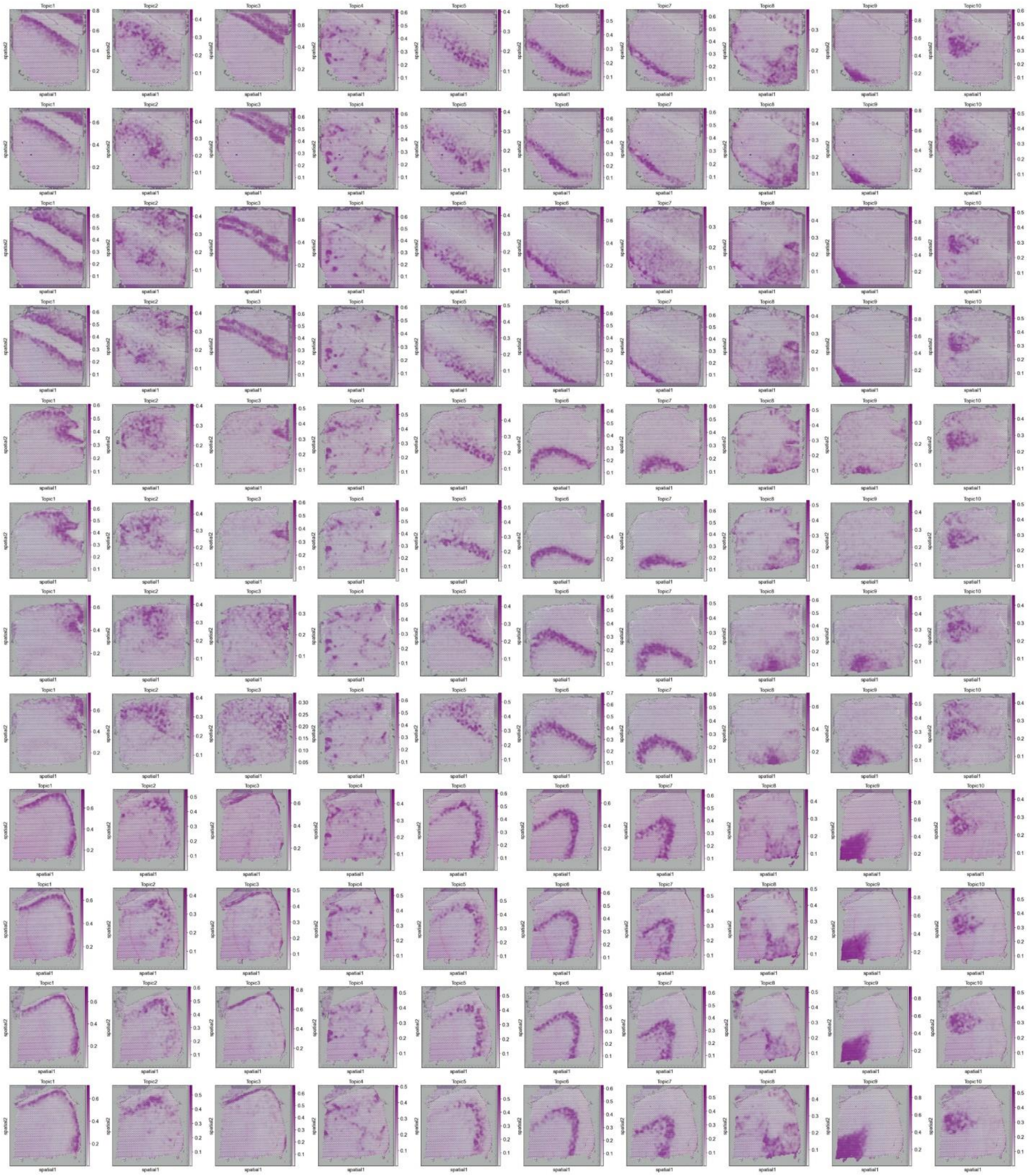

Figure S19: Topic proportions returned by STAMP for the 10x Genomics Visium DLPFC data (from top to bottom, 151507, 151508, 151509, 151510, 151669, 151670, 151671, 151672, 151673, 151674, 151675, 151676).

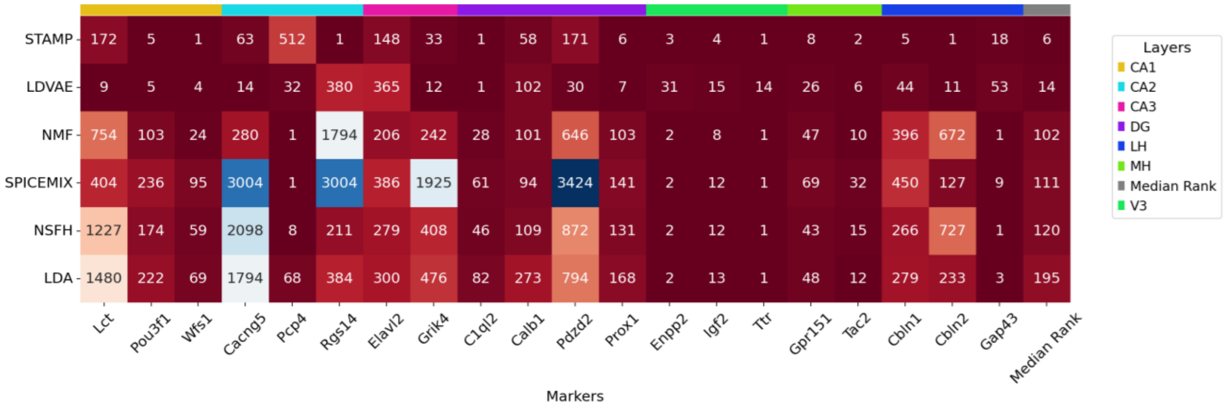

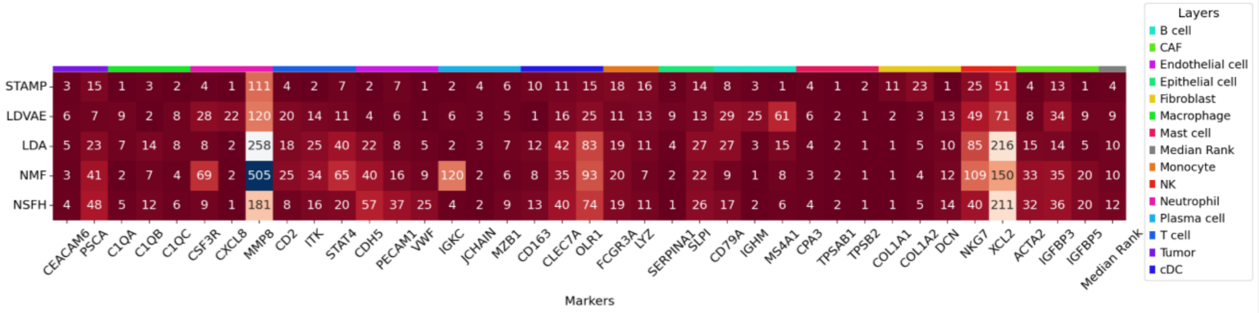

Figure S20A: Ranking of selected marker genes in the associated gene modules of topics found by each method for the Slide-seq V2 mouse brain (top) and Nanostring SMI NSCLC (bottom) data. STAMP outperformed the other methods at marker gene identification.

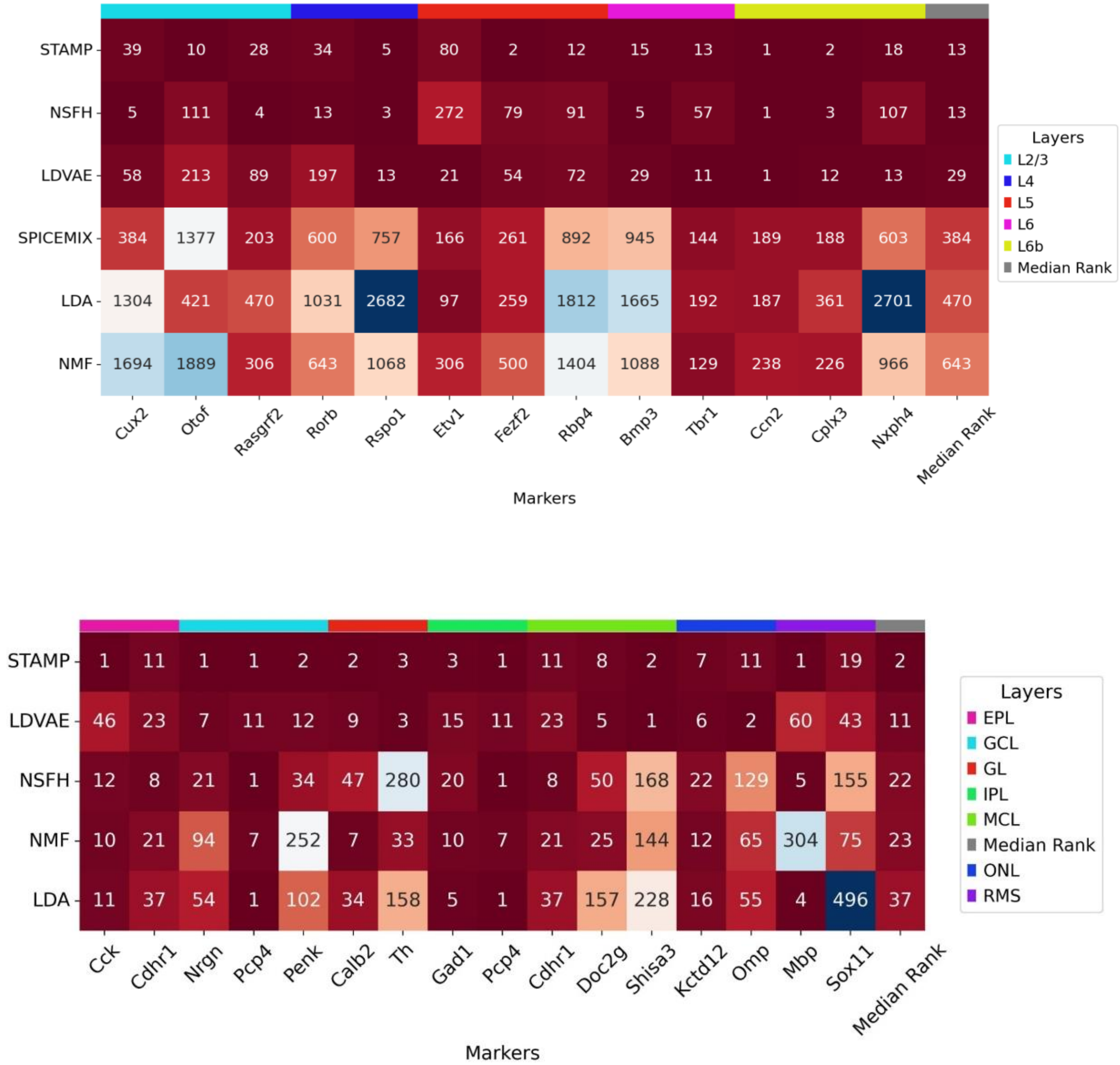

Figure S20B: Ranking of selected marker genes in the associated gene modules of topics found by each method for the 10x Genomics Visium mouse brain (top) and Stereo-seq mouse olfactory bulb (bottom) data. STAMP outperformed the other methods at marker gene identification.

Supplementary Note 1: Technical details

Graphical model

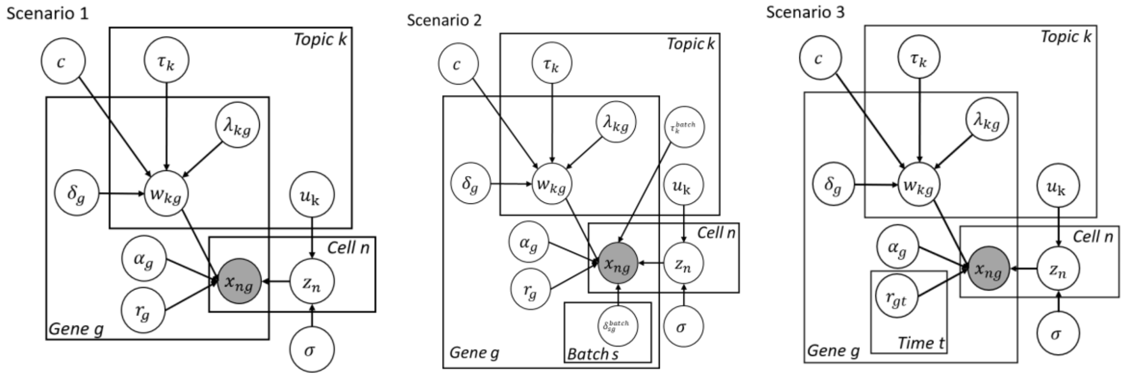

Figure S21: Graphical models of the three different modes of STAMP. Scenario 1 refers to single sample STAMP. Scenario 2 refers to multi sample STAMP, where we have two additional terms, δ_{sg}^{batch} and τ_k^{batch}, which quantify the effect of batches. Scenario 3 refers to STAMP with time-series data. Note that in the third scenario, $w_{kg} \in R^T$, while $w_{kg} \in R^1$ for the first scenario. The length scale random variable of the Gaussian process is omitted in Scenario 3 as it is fixed at 1.

Inference for scenario 2 and 3

Inference for scenario 1 is described in detail in the Methods section. For the other two scenarios, we also use black box variational inference. In scenario 2, the inference is almost identical to the first scenario, except that we have extra two terms τ_k^{batch} and δ_{sg}^{batch} which needs to be estimated. Similarly, we used the mean-field variational family $q(\tau_k^{batch})$ and $q(\delta_{sg}^{batch})$ to approximate the true posterior.

In scenario 3, our Gaussian process spans across timepoints which are small (8 in our datasets) compared to other methods such as NSFH which spans across spatial locations (>3000). Therefore, we do not make use of Sparse Gaussian Processes which learns inducing points. The term that is different here is $w_{kg} \in R^T$, compared to scenario 1. Here, we use the multivariate normal $q(w_{kg})$ to approximate the posterior. In particular, we use the Cholesky decomposition to learn this term.

Simplified Graph Convolutions

STAMP uses a simplified graph convolutional (SGC) neural network (Wu, et al. and Frasca, et.al [1,2]), which is a linearization of the conventional graph-convolutional neural network (Kipf, et al.[3]). Here we describe the SGC architecture (which is also provided in the Supplementary Note 2).

A lth layered graph convolutional network can be expressed as

$$\tilde{A} = A + I$$
$$H^{(0)} = X$$
$$S = \tilde{D}^{-\frac{1}{2}} \tilde{A} \tilde{D}^{-\frac{1}{2}}$$
$$H^{(l)} = \sigma\left(SH^{(l-1)}W^{(l)}\right)$$

where X is the gene expression matrix and σ is a nonlinear activation function such as ReLU. I is the identity matrix and $\widetilde{D}$ is the degree of $\tilde{A}$. S is also known as the symmetric normalized adjacency matrix.

Wu et al.[2] hypothesized that the success of GCNs is due to local averaging and proposed a simplified graph convolution that linearizes the graph convolutional network by removing the nonlinear activation functions σ, which leads to

$$\begin{aligned} H^{(l)} &= SH^{(l-1)}W^{(l)} \\ &= SSH^{(l-2)}W^{(l-1)}W^{l} \\ &= S \dots SSH^{(0)}W^{(1)} \dots W^{(l)} \\ &= S^{l}XW \end{aligned}$$

where the weights $W^{(1)} \dots W^{(l)}$ can be combined into a single weight W.

The simplified graph convolution is more efficient than the conventional graph convolutional neural network where we can precompute the $S^{l}X$, which is a smoothing of the gene expression with a normalized adjacency matrix. This aids in scalability, as we can now train simple graph convolutions as multilayer perceptrons with mini-batched training. Regular graph neural networks such as graph convolutional network and graph attention networks are unable to do so as we need to fit the whole dataset in the GPU memory during training, therefore limiting their scalability with large datasets.

Given that time efficiency is a crucial factor when dealing with large spatial transcriptomic data, we incorporated the simplified graph convolution into STAMP to smooth the gene expression matrix X before propagating it through an MLP (Figure S25). However, we observed that relying solely on the smoothed expression matrix resulted in over-smoothed latent topics. We hypothesized that this was due to their existing both spatial and non-spatial pattern in the dataset. Therefore, we concatenated the smoothed matrix with the original gene expression matrix. Note that the S here is not the batch label but the smoothing matrix.

Training details

The inputs to the neural network are the original gene expression matrix and the smoothed matrix as described in the previous section:

$$\begin{aligned} \tilde{A} &= A + I \\ S &= \widetilde{D}^{-\frac{1}{2}}\, \tilde{A}\, \widetilde{D}^{-\frac{1}{2}} \\ z_u\,, z_\sigma &= NN\ ([X\ , SX, \dots S^{l}X]) \\ q(z_n\ |X, A) &= softmax(Normal(z_u, z_\sigma)) \end{aligned}$$

where l is the number of layers. We used l = 1 for all experiments. We trained all of our models with a clipped AdamW optimizer[4] implemented in Pyro where we clipped gradients that were larger than 1. We used a batch-size of 256 with a learning rate of 0.01. For the mouse olfactory bulb and mouse embryo datasets, the smallest data was smaller than 5% of the largest dataset. Therefore, to ensure equal representation in each mini batch, we made use of a weighted sampler which drew samples inversely proportional to the size of each dataset. We used an early stopping strategy where we stopped after the model loss plateaued for 30 epochs.

We found that training the covariance variational parameters $q(u_k)$ and $q(\sigma)$ with the rest of the parameters led to sub-optimal topics where many topics were identical. Therefore, we only started to train these parameters after the model loss plateaued for 5 epochs.

Network architecture

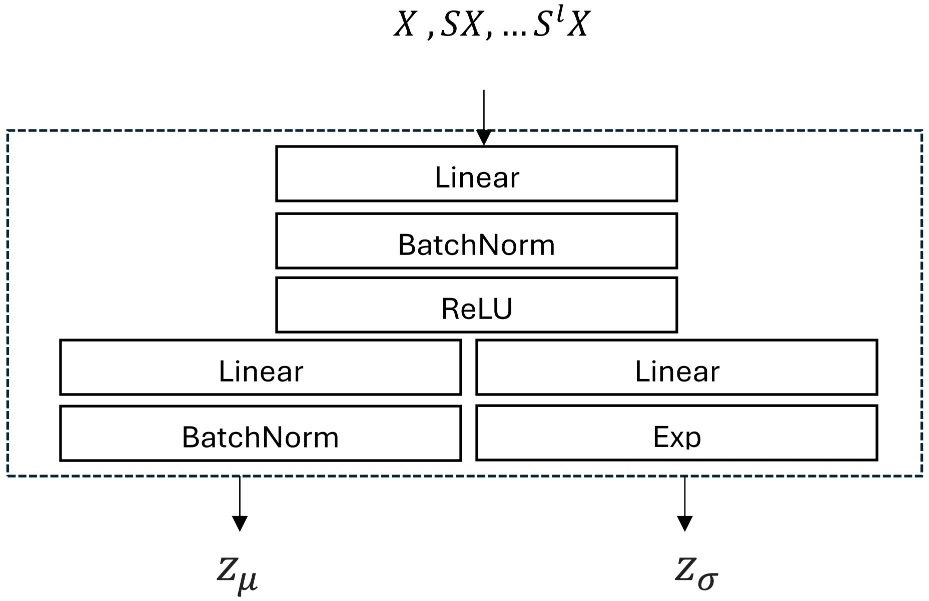

Figure S22: Neural network architecture.

Supplementary Note 2: Simulation study

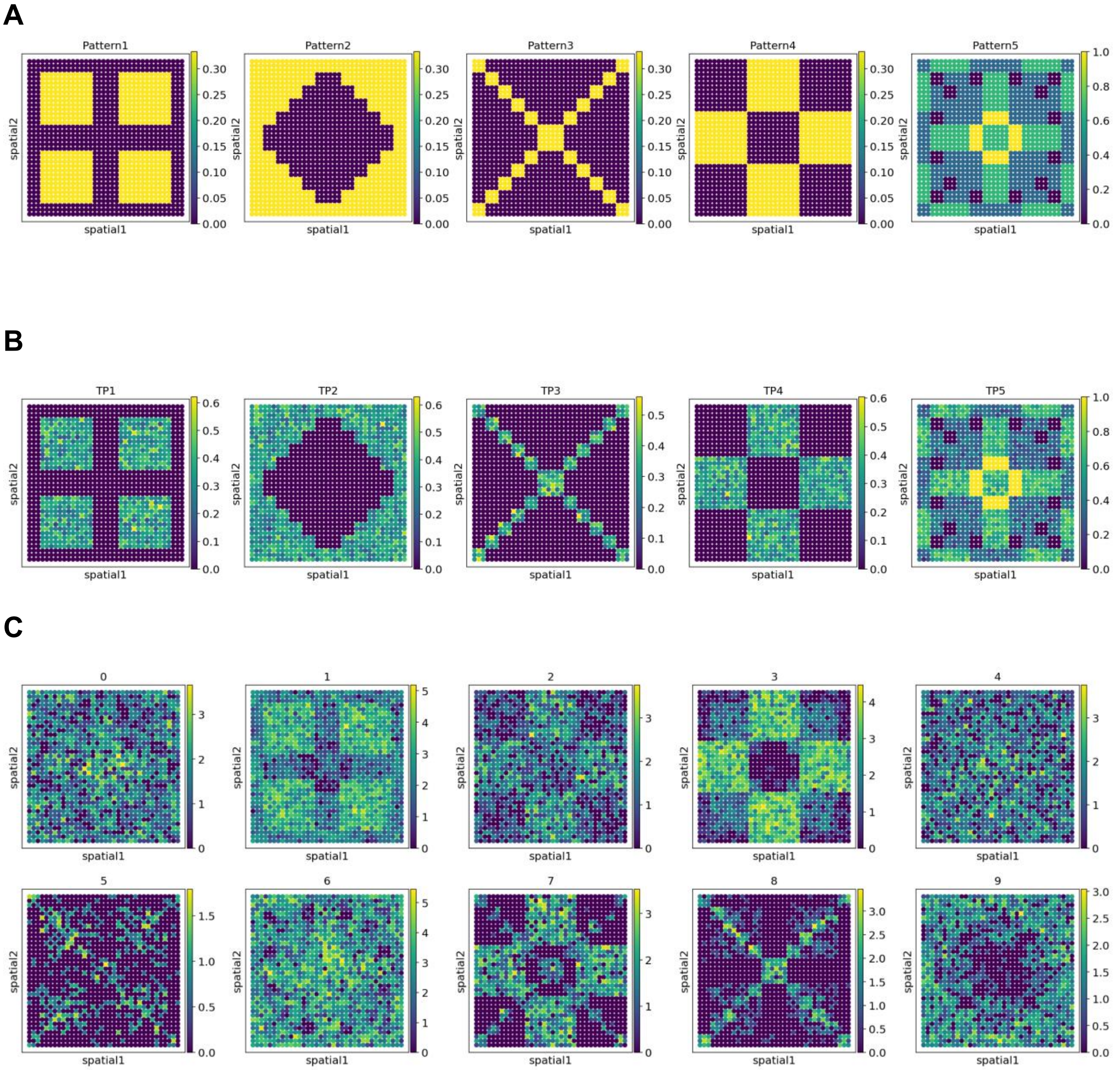

Figure S23: Simulated spatial count data. **A** Pattern for simulation **B** Ground truth topic proportion **C** Example of simulated gene counts. Each gene is made up of a combination of different topics and some background noise.

The dataset was simulated as follows:

$$z_{nk} \sim Dirichlet(10 * Pattern_1 + \epsilon, \dots, 10 * Pattern_k + \epsilon)$$
$$u_{kg} \sim Bernoulli(0.25)$$
$$t_{kg} \sim LogNormal(0, 1)$$
$$\epsilon \sim LogNormal(-1, 1)$$

$$w_{kg} \sim Dirichlet(u_{kg} * t_{kg} + \epsilon)$$
$$ls_n \sim Uniform(2000, 10000)$$
$$\alpha_g \sim Exponential(0.2)$$
$$x_{ng} \sim Gamma(\alpha_g, \alpha_g/(z_{nk} * \mathrm{w}_{kg} * l_n))$$
$$y_{ng} \sim Poisson(x_{ng})$$

where Z_{nk} is the simulated ground truth topic proportion of topic k in cell or spot n. $Pattern_1, \ldots, Pattern_k$ are the k simulated spatial patterns or topics. u_{kg} is the usage of gene g in topic k, drawn from a Bernoulli distribution. For gene g that is activated in topic k, its expression is given by t_{kg} that is generated from a lognormal distribution. Therefore, the expression level of each gene module per topic is given by $u_{kg} * t_{kg}$. ϵ is the background noise. The ground truth gene module w_{kg} is then generated by sampling through a Dirichlet distribution. y_{ng} is the spatial count data simulated from a Gamma Poisson distribution where the dispersion term α_g is generated from an exponential distribution, and the mean is given by $z_{nk} * \mathrm{w}_{kg} * l_n$.

We ran STAMP, LDA, LDVAE, NMF, NSFH, and SpiceMix on the simulated data. Among the tested methods, LDVAE and NMF showed the poorest performance, being only able to uncover factor 1. NSFH, STAMP, LDA and SPICEMIX were able to recover all the ground truth topics. NSFH and STAMP, in particular were able to accurately recover the different topics. We next compared their performance using quantitative metrics, namely Pearson correlation coefficient on both the topic proportions and gene modules. The former measures the correlation between the estimated topic proportion and the ground truth topic proportion, while the latter measures the correlation between the estimated gene modules and the ground truth gene modules. In concordance with the visual inspection, LDVAE scored the lowest, and STAMP scored similar to NSFH on both metrics.

STAMP

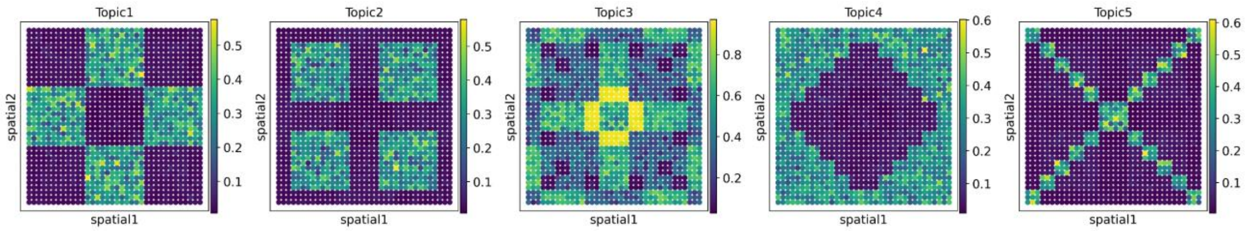

SPICEMIX

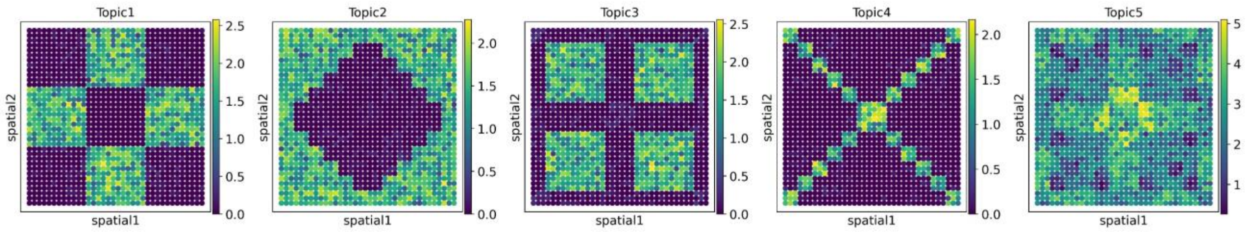

LDVAE

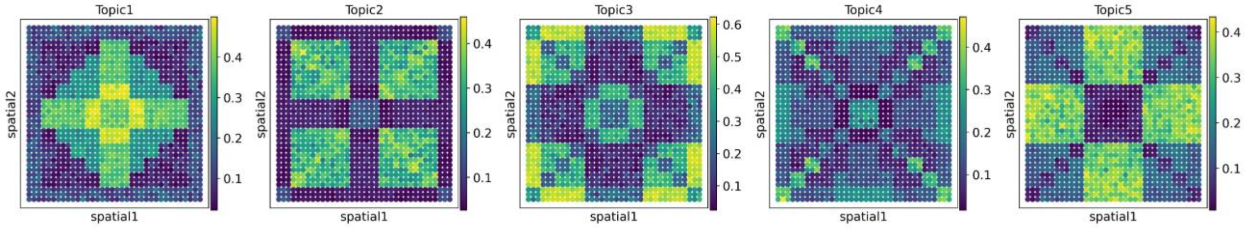

NMF

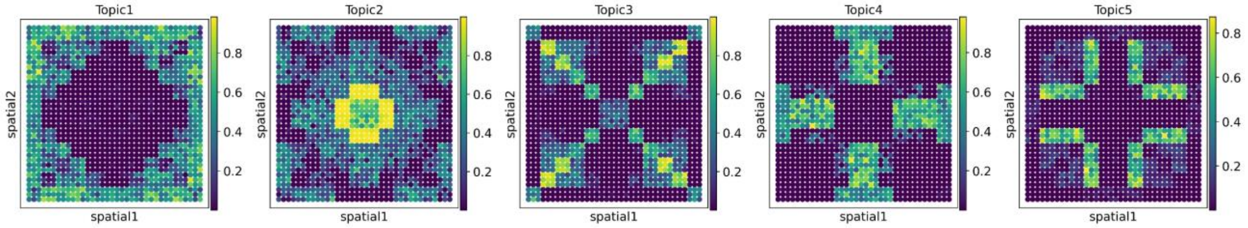

LDA

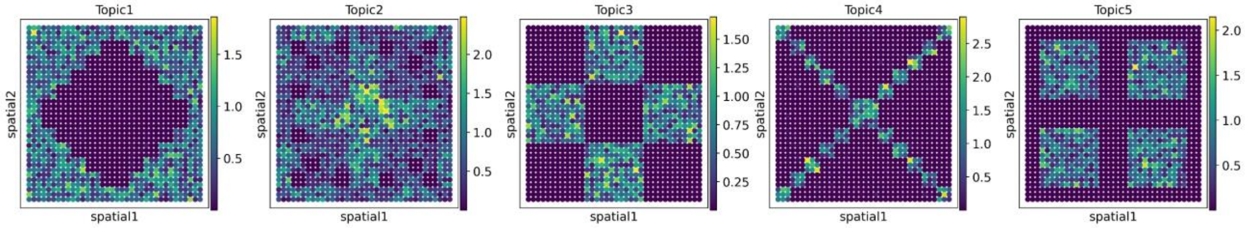

NSFH

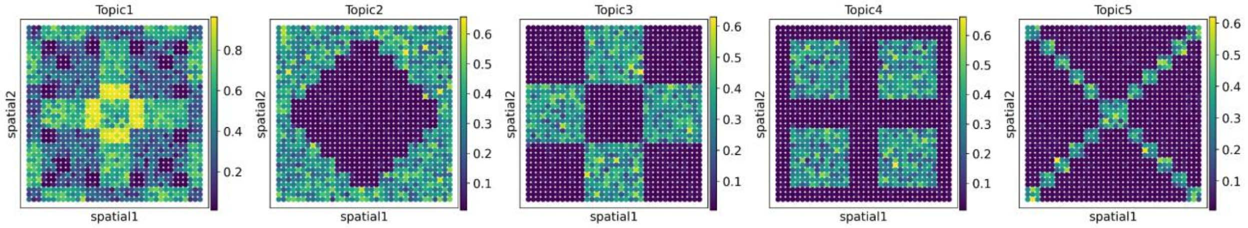

Figure S24: Topics returned by the different methods for the simulated data.

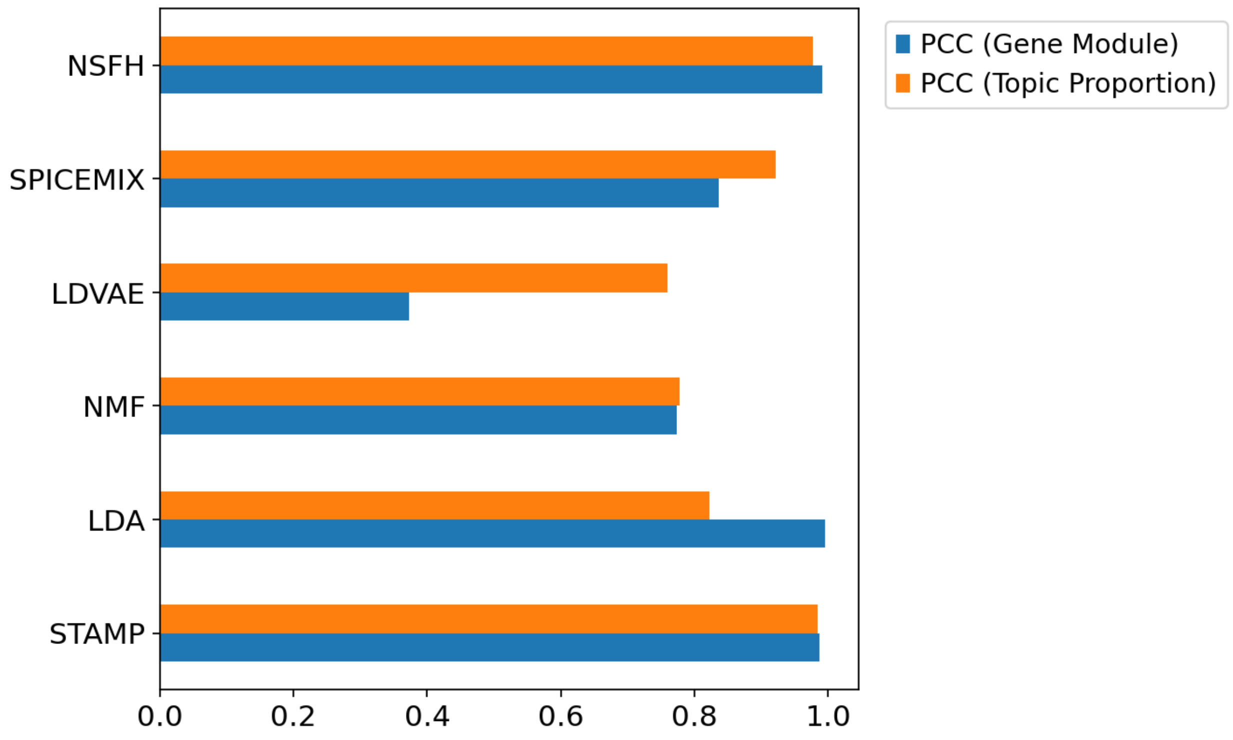

Figure S25: Pearson correlation score of both gene modules and topic proportion with the ground truth topic proportions and gene modules, respectively.

Supplementary Tables

Table S1: The top two results returned by the DISCO toolkit. DISCO in general was accurate at identifying the cell type signatures.

Topics	Pval	Or	Name	Gene
Epithelial cell (Topic 1)	0	51.959	AT2 vs All others in lung	CHI3L1,LAMP3,CXCL2,DMBT1,SLPI,CXCL17,EFNA1,ADGRF5,AQP3,KRT7,SERPINA1,ICAM1
	0	50.285	Goblet cell vs Airway basal cell in lung	SLPI,CXCL17,CLU,LTF,CXCL2,KRT7,ADGRF5,SERPINA1
Endothelial cell (Topic 2)	0	70.785	Venous EC vs All others in lung	TIE1,ADGRL4,ACKR1,RGS5,SPARCL1,GPX3,RAMP3,TEK,ESAM,VWF,FLT1,CLEC14A,CDH5,RAMP2,PECAM1,CD34,ADGRL2,COL4A1
	0	64.018	Capillary EC vs All others in lung	TIE1,ADGRL4,ADGRL2,SPARCL1,RAMP3,TEK,ESAM,VWF,FLT1,COL4A1,CLEC14A,CDH5,RAMP2,PECAM1,CD34,RGS5,GPX3,KDR
Mast cell (Topic 3)	0	86.429	Mast cell vs All others in lung	RGS1,RGS2,IL1RL1,CPA3,KIT,HPGDS,PTGS1,TPSB2,TPSAB1,LIF,CD69,IL18R1,ADGRE2,CD33,AREG
T/NK (Topic 5)	0	77.249	GZMK CD8 T cell vs All others in lung	CD2,CD8A,DUSP2,TIGIT,FYB1,GZMK,GZMA,CD69,CCL5,CST7,ITK,STAT4,CD28,PTPRCAP,IL2RB,IL7R
	0	73.825	GZMB CD8 T cell vs All others in lung	CD2,CD8A,GZMA,CCL5,CST7,IL2RB,GZMK,STAT4,ITK,TIGIT,CD69,DUSP2,FYB1,IL7R,PTPRCAP
Fibroblast (Topic 6)	0	94.45	ADAMDEC1+ADAM28+ fibroblast vs All others in lung	COL3A1,COL5A2,COL6A3,PDGFRA,COL1A2,COL5A1,CDH11,COL1A1,VCAN,MEG3,FN1,ACTA2,LUM,TAGLN,COL8A1,THBS1
Macrophage (Topic 7)	0	190.913	LYVE1 macrophage vs All others in lung	C1QA,C1QC,C1QB,FCER1G,MERTK,SELENOP,CD14,CD74,HLA-DRB1,HLA-DQA1,HLA-DPA1,GPNMB,MS4A4A,MRC1,CD163,CD68,PSAP,GLUL,CCL18
	0	106.701	M1 macrophage vs All others in lung	C1QA,C1QC,C1QB,FCER1G,GLUL,CD14,CD74,HLA-DRB1,HLA-DQA1,HLA-DPA1,GPNMB,MS4A4A,MRC1,PSAP,CD163,CD68,CCL18
Tumor interior (Topic 8)	0	32.913	Goblet cell vs All others in lung	S100P,PSCA,KRT8,KRT19,CEACAM6,KRT17
	0	29.101	Airway basal cell vs All others in lung	SLC2A1,KRT8,KRT19,KRT17,S100P,ANXA2,IER3,CEACAM6,S100A6
Tumor edge (Topic 9)	0	71.899	Goblet cell vs All others in lung	PIGR,S100P,AGR2,MMP7,MSMB,KRT8,KRT19,EPHA2,GSTP1
	0	38.589	Goblet cell vs Airway basal cell in lung	PIGR,MSMB,LYZ,MMP7,S100P,AGR2
cDC/Monocyte (Topic 10)	0	401.837	LYVE1 macrophage vs All others in lung	FCER1G,CSF1R,CD74,HLA-DRA,HLA-DRB1,HLA-DQA1,HLA-DPA1,HLA-DPB1,MRC1,CD163,CLEC7A,CLEC10A,LYZ,CSF2RA,TLR2,RGS1,FCGR3A,OLR1,CIITA,RGS2

	0	259.446	cDC2 vs All others in lung	FCER1G,RGS1,CSF1R,CD74,HLA-DRA,HLA-DRB1,HLA-DQA1,HLA-DPA1,HLA-DPB1,CSF2RA,CLEC7A,LYZ,CLEC10A,CIITA,OLR1,MRC1,RGS2,TLR2,CD163
Fibroblast (Topic 11)	0	120.912	CFD+MGP+ fibroblast vs All others in lung	MXRA8,PDGFRA,PTGDS,MGP,LUM,DCN,MMP2,COL6A1,COL6A2,COL6A3,COL3A1,COL1A1,RARRES1,BGN,TIMP1,IGFBP7,IGF1,COL14A1
B cell (Topic 12)	0	113.651	Memory B cell vs All others in lung	CD52,FCRLA,SELL,CD74,LTB,MS4A1,TCL1A,IGHD,IGHM,CD19,P2RX5,TNFRSF13B,CD79A,CD37,VPREB3,CD83,CIITA
	0x	63.434	Naive B cell vs All others in lung	FCRLA,TCL1A,IGHM,CD19,CD79A,VPREB3,MS4A1,IGHD,P2RX5,CD37,CD74,LTB,CD52,IRF4
CAF (Topic 13)	0	83.828	Vascular smooth muscle cell vs All others in lung	IGFBP5,IGFBP7,PDGFRB,CALD1,BGN,TAGLN,ACTA2,MYH11,MYL9,NOTCH3,COL6A2,RGS5,COL1A1,COL18A1,COL4A2,COL4A1
	0	70.86	Pericyte vs All others in lung	RGS5,IGFBP7,PDGFRB,CALD1,BGN,COL4A1,COL4A2,MYL9,COL5A3,NOTCH3,COL6A2,TAGLN,ACTA2,COL18A1,MYH11,IGFBP5
Plasma cell (Topic 14)	0	175.724	Plasma cell vs All others in lung	IGKC,CD38,JCHAIN,MZB1,IRF4,CD27,IGHG2,IGHA1,IGHG1,IGHM,TNFRSF17,CD79A,XBP1
	0	66.229	Plasma cell vs Memory B cell in lung	MZB1,JCHAIN,XBP1,IGHM,IGHA1,TNFRSF17,IGHG1,CD27,CD38,ITGA6,IRF4,IGKC,IGHG2
Tumor (Topic 15)	0	165.291	Goblet cell vs All others in lung	TACSTD2,S100P,CLDN4,PSCA,LCN2,MMP7,KRT7,KRT8,KRT18,KRT19,CEACAM6,EPHA2,KRT17
	0	57.272	Airway basal cell vs All others in lung	EPHA2,TACSTD2,CLDN4,KRT8,KRT18,KRT19,KRT17,S100P,KRT7,LCN2,CEACAM6,S100A6

Table S2: Detailed information on the technology, number cells and genes of employed datasets before and after preprocessing.

Figure	Technology	Tissue	Number of cells (original)	Number of genes (original)	Number of cells (pre-processed)	Number of genes (pre-processed
2A	Slideseq-v2	Mouse brain	41,770	23,264	39,220	6,000
3A	Spatial Molecular Imager	Human small-lung cell cancer	98,002	960	93,206	600
4A	Visium	Mouse brain anterior	3,289	32,285	6,110	2,000
		Mouse brain posterior	2,825	32,285		
5A	Stereo-seq	Mouse olfactory bulb	107,416	26,145	148,047	6,000
	Visium		1,185	32,835		

	Slide-seq V2		47,249	22,167		
5D	Visium	Human DLPFC	47,681	33,538	47,329	4,000
6A	Stereo-seq	Mouse embryo E9.5 to E16.5	520,815	23,761	510,312	2,000

Supplementary References

1. Frasca, F. *et al.* SIGN: Scalable Inception Graph Neural Networks. Preprint at https://doi.org/10.48550/arXiv.2004.11198 (2020).
2. Wu, F. *et al.* Simplifying Graph Convolutional Networks. in *Proceedings of the 36th International Conference on Machine Learning* 6861–6871 (PMLR, 2019).
3. Kipf, T. N. & Welling, M. Semi-Supervised Classification with Graph Convolutional Networks. Preprint at https://doi.org/10.48550/arXiv.1609.02907 (2017).
4. Loshchilov, I. & Hutter, F. Decoupled Weight Decay Regularization. Preprint at https://doi.org/10.48550/arXiv.1711.05101 (2019).
