# Supplementary material for: Interpretable spatially aware dimension reduction of spatial transcriptomics with STAMP
Source: Nat Methods. 2024 Oct 15;21(11):2072–83. doi: 10.1038/s41592-024-02463-8 (PMC11541207; doi:10.1038/s41592-024-02463-8)
Supplement: Supplementary file 2 — Reporting Summary [file 41592_2024_2463_MOESM2_ESM.pdf]

nature portfolio

Corresponding author(s): Jinmiao Chen

Last updated by author(s): 22/08/24

Reporting Summary

Nature Portfolio wishes to improve the reproducibility of the work that we publish. This form provides structure for consistency and transparency in reporting. For further information on Nature Portfolio policies, see our Editorial Policies and the Editorial Policy Checklist.

Statistics

For all statistical analyses, confirm that the following items are present in the figure legend, table legend, main text, or Methods section.

n/a	Confirmed	
☐	☒	The exact sample size (*n*) for each experimental group/condition, given as a discrete number and unit of measurement
☐	☒	A statement on whether measurements were taken from distinct samples or whether the same sample was measured repeatedly
☐	☒	The statistical test(s) used AND whether they are one- or two-sided<br>*Only common tests should be described solely by name; describe more complex techniques in the Methods section.*
☐	☒	A description of all covariates tested
☐	☒	A description of any assumptions or corrections, such as tests of normality and adjustment for multiple comparisons
☐	☒	A full description of the statistical parameters including central tendency (e.g. means) or other basic estimates (e.g. regression coefficient) AND variation (e.g. standard deviation) or associated estimates of uncertainty (e.g. confidence intervals)
☐	☒	For null hypothesis testing, the test statistic (e.g. *F*, *t*, *r*) with confidence intervals, effect sizes, degrees of freedom and *P* value noted<br>*Give P values as exact values whenever suitable.*
☐	☒	For Bayesian analysis, information on the choice of priors and Markov chain Monte Carlo settings
☒	☐	For hierarchical and complex designs, identification of the appropriate level for tests and full reporting of outcomes
☒	☐	Estimates of effect sizes (e.g. Cohen's *d*, Pearson's *r*), indicating how they were calculated

Our web collection on statistics for biologists contains articles on many of the points above.

Software and code

Policy information about availability of computer code

Data collection	No software was used for the data collection
Data analysis	scTM v0.1.3(https://github.com/JinmiaoChenLab/scTM), pytorch v2.0.3(https://github.com/pytorch/pytorch), pyro v1.8.4(https://github.com/pyro-ppl/pyro) were used for designing the algorithm.<br>Sklearn v1.2.1(https://github.com/scikit-learn/scikit-learn), Spicemix v1.0.0(https://github.com/ma-compbio/SpiceMix), nsf vN.A. (https://github.com/willtownes/nsf-paper), scVI v1.0.3 (https://github.com/scverse/scvi-tools) were used for benchmarking.<br>Scanpy v1.9.1(https://github.com/scverse/scanpy), anndata v0.9.1(https://github.com/scverse/anndata), Seurat v4.3.0(https://github.com/satijalab/seurat), glmGamPoi v1.12.2(https://github.com/const-ae/glmGamPoi), squidpy v1.2.2(https://github.com/scverse/squidpy) were used for preprocessing and post-processing of spatial data, STAGATE vN.A. (https://github.com/QIFEIDKN/STAGATE), GraphST vN.A.(https://github.com/JinmiaoChenLab/GraphST), spatialPCA vN.A.(https://github.com/shangll123/SpatialPCA). DeepST v N.A.(https://github.com/JiangBioLab/DeepST)

For manuscripts utilizing custom algorithms or software that are central to the research but not yet described in published literature, software must be made available to editors and reviewers. We strongly encourage code deposition in a community repository (e.g. GitHub). See the Nature Portfolio guidelines for submitting code & software for further information.

Data

Policy information about availability of data

All manuscripts must include a data availability statement. This statement should provide the following information, where applicable:

- Accession codes, unique identifiers, or web links for publicly available datasets
- A description of any restrictions on data availability
- For clinical datasets or third party data, please ensure that the statement adheres to our policy

We analyzed a total of 8 spatial transcriptomics datasets for evaluation of latent topics and gene modules. Publicly available data were downloaded from the following websites

1. Mouse hippocampus, Slide-seqV2
The data is downloaded from https://github.com/satijalab/seurat-data.
2. Human non-small cell lung cancer (NSCLC) Data, SMI
The processed Giotto object was downloaded from https://nanostring.com/products/cosmx-spatial-molecular-imager/nsclc-ffpe-dataset.
3. Mouse brain anterior and posterior Data, Visium
The count matrix and spatial data can be downloaded from https://www.10xgenomics.com/resources/datasets. The stitched h5ad can be found in the accompanying zenodo file at https://zenodo.org/records/10988053
4. Mouse olfactory bulb data, Stereo-seq
We obtained the Stereo-seq data from https://db.cngb.org/stomics/mosta/download/. The file to download is Mouse_olfa_S1.h5ad
5.Mouse olfactory bulb data Slide-seq V2
The Slide-seq V2 data was obtained from https://singlecell.broadinstitute.org/single_cell/study/SCP815/highly-sensitive-spatial-transcriptomics-at-near-cellular-resolution-with-slide-seqv2#study-download.
6. Mouse olfactory bulb data 10x Genomics Visium
The 10x Genomics Visium data was obtained from https://www.10xgenomics.com/datasets/adult-mouse-olfactory-bulb-1-standard.
7. Human DLPFC, Visium
The count matrix can be found at https://github.com/LieberInstitute/spatialDLPFC.
8. Mouse Embryo Data E9.5 to E16.5, Stereo-seq
The count matrix and spatial data can be downloaded from https://db.cngb.org/stomics/mosta/download/. The files to download are Mouse_embryo_all_stage.h5ad and E10.5_E1S2.MOSTA.h5ad.
9. MsigDB gene sets
The gene sets can be downloaded from https://www.gsea-msigdb.org/gsea/msigdb

All of the processed data can be found at https://zenodo.org/records/10988053.

Research involving human participants, their data, or biological material

Policy information about studies with human participants or human data. See also policy information about sex, gender (identity/presentation), and sexual orientation and race, ethnicity and racism.

Reporting on sex and gender	N.A.
Reporting on race, ethnicity, or other socially relevant groupings	N.A.
Population characteristics	N.A.
Recruitment	N.A.
Ethics oversight	N.A.

Note that full information on the approval of the study protocol must also be provided in the manuscript.

Field-specific reporting

Please select the one below that is the best fit for your research. If you are not sure, read the appropriate sections before making your selection.

☒ Life sciences ☐ Behavioural & social sciences ☐ Ecological, evolutionary & environmental sciences

For a reference copy of the document with all sections, see nature.com/documents/nr-reporting-summary-flat.pdf

Life sciences study design

All studies must disclose on these points even when the disclosure is negative.

Sample size	We used 8 publicly available data in the manuscript.

Data exclusions	We removed spots and genes by applying the standard preprocessing steps such as removing lowly expressed genes and spots.
Replication	N.A. Our experiments did not aim to uncover any mechanistic or intervention effect. Instead, we benchmarked our proposed methodology against competing methods with different datasets, across different technologies
Randomization	N.A. Our experiments did not aim to uncover any mechanistic or intervention effect, hence we did not need any controls.
Blinding	N.A. Our experiments did not involve human participants and response.

Reporting for specific materials, systems and methods

We require information from authors about some types of materials, experimental systems and methods used in many studies. Here, indicate whether each material, system or method listed is relevant to your study. If you are not sure if a list item applies to your research, read the appropriate section before selecting a response.

Materials & experimental systems

n/a	Involved in the study
☒	☐ Antibodies
☒	☐ Eukaryotic cell lines
☒	☐ Palaeontology and archaeology
☒	☐ Animals and other organisms
☒	☐ Clinical data
☒	☐ Dual use research of concern
☒	☐ Plants

Methods

n/a	Involved in the study
☒	☐ ChIP-seq
☒	☐ Flow cytometry
☒	☐ MRI-based neuroimaging
